# Spatio-temporal evolution of eutrophication and water quality in the Turawa dam reservoir, Poland

**DOI:** 10.1038/s41598-023-36936-1

**Published:** 2023-06-19

**Authors:** Bogna Buta, Mirosław Wiatkowski, Łukasz Gruss, Paweł Tomczyk, Robert Kasperek

**Affiliations:** Institute of Environmental Engineering, Wrocław University of Environmental and Life Sciences, 50-363 Wrocław, Poland

**Keywords:** Environmental monitoring, Environmental impact

## Abstract

The objectives of the article are: to assess spatio-temporal evolution of eutrophication and water quality of the Turawa dam reservoir, located in south-western Poland on the Mała Panew River; to identify location and relationship between potential sources of physicochemical pollution related to the progressing process of eutrophication; and to determine trophic status and water quality indices of the selected research object. The analysis (Mann–Whitney U test, PCA, HCA, Spearman correlation matrix) showed a high susceptibility of the reservoir to eutrophication processes, especially due to the influence of dangerous loads of compounds emerging from areas with high tourist intensity and pollutants flowing from the Mała Panew River. The parameters deteriorating the ecological status were TP, DO, BOD_5_, and COD. Considering the cumulative results of water quality indices for the period 1998–2020, the average water quality was in classes II or III. A noticeable deterioration appeared in water quality for the years 2016–2020, which proves the progressing eutrophication in the Turawa reservoir. In 1998–2020, the reservoir was classified as eutrophic or mesoeutrophic based on the calculated three trophic status indices. This article would help in developing a strategy for dealing with water blooms, a reliable system for monitoring pressures causing eutrophication, and optimal technologies for the reconstruction of multifunctional reservoirs.

## Introduction

Water is used for various purposes (agriculture, industry, services, household use, recreation, shipping, life processes of organisms, etc.) and participates in shaping hydromorphological and geomorphological processes^1^. However, its resources per capita are decreasing on a global scale (comparing 2018 to 1962, these resources decreased by 57.8%, i.e., amounting to 5658 and 13,407 m^3^ per capita, respectively) and are diversified in different regions of the world (smallest in the northern part of Africa with 256 m^3^ per capita in 2015 and largest in South America and Oceania with approximately 30,000 m^3^ per capita)^2,3^. Therefore, maintaining its good quality is an important socio-economic issue and will become increasingly important in the future^4,5^. Water quality can be affected by numerous natural (e.g., river erosion and geology) and anthropogenic factors (e.g., agriculture, industry, and households)^6,7^. Water has various forms, but humans dominantly use freshwater, i.e., groundwater and surface water (rivers, water reservoirs, and wetlands), which only account for approximately 1% of the total water resources in the world^8^.

Since surface water bodies can perform a number of functions, the water accumulated in reservoirs is of particular importance, e.g., flood protection, hydropower use, bathing areas, fishing, recreation, natural habitat, source of drinking water, and use for other purposes (e.g., agricultural, economic)^9–11^. However, these functions may be disturbed by various unfavorable processes, such as eutrophication, which is caused by the influx of large amounts of organic substances, particularly those rich in phosphorus and nitrogen compounds, and most often has an agricultural origin^12–14^. Eutrophication increases the fertility of the reservoir, which in turn cause the development of undesirable cyanobacteria, diatoms, and green algae, which manifest themselves in the form of the water bloom^15,16^. These organisms use oxygen for the processes of photosynthesis and quickly multiply their biomass^17^. Their excessive growth causes a significant deterioration in water quality (especially, oxygen and light conditions), and the reservoirs themselves cannot perform the assumed functions^18^. Eutrophicated water may also exhibit toxic properties due to the release of metabolic products from cyanobacteria^19^. Studies have shown that the factors limiting the size of blooms are phosphorus and nitrogen compounds, but their role varies depending on the season, region, or specificity of water reservoirs^20,21^. Typically, phosphorus is the limiting factor for eutrophication in spring, and nitrogen in summer and autumn, due to the specificity of temperatures and meteorological conditions^22,23^. The main source of phosphorus in water bodies is its release from sediments^24^, and in the case of nitrogen, from denitrification processes^25^.

A study of over 2,000 water bodies showed that 63.1% of them were eutrophic and were mainly concentrated in eastern Asia, northern Africa, and central north and south-eastern North America^26^. Eutrophication is a significant problem, especially in inland waters, and its frequency, intensity, and scale are increasing every year on a global scale^20^. Owing to the noticeable eutrophication in water bodies around the world, it is being researched. For example, in the following sites: Lake Erhai and Three Gorges Reservoir in China^27,28^, Englishmen Lake in Spain^29^, Lake Tana in Ethiopia^30^, Lake Cedrino in Italy^31^, Cointzio reservoir in Mexico^32^, Lake Taupo in New Zealand^33^, Lake Erie in the United States^34^, Lake Ypacaraí in Paraguay^35^, Keban reservoir in Turkey^36^, and Anavilundawa in Sri Lanka^37^. Climate change may have intensified eutrophication process by altering temperature and humidity, manifesting in more frequent occurrence of hydrological extreme phenomena, such as droughts and floods (higher temperatures and persistently low water levels rapidly influx organic matter and favor development of algae, responsible for eutrophication)^38–40^. Eutrophication in reservoirs itself may accelerate the increase in air temperature on a larger scale due to the emissions of greenhouse gases, mainly carbon dioxide (CO_2_) and methane (CH_4_), produced by algae^41^.

The objectives of the article are: (I) to assess spatio-temporal evolution of eutrophication and water quality in the Turawa dam reservoir (capacity of approximately 110 million m^3^)^42^, located in the south-western Poland on the Mała Panew River (right tributary of the Odra River), (II) to identify the location and relationship between potential sources of physicochemical pollution related to the progressing of eutrophication^43^ (parameters characterizing the amount of organic matter, enrichment in nutrients, and oxygen conditions in water were analysed), and (III) to determine trophic status and water quality indices in the selected research object. In this work, a number of multivariate techniques and tools were used for statistical analysis of the results^44^, i.e., Principal Component Analysis (PCA), Hierarchical Cluster Analysis (HCA), and a correlation matrix using Spearman's rank correlation coefficients to determine the variability of data between parameters and between points. Maps were also prepared to demonstrate the spatial variability and distribution of the considered physicochemical parameters of water (pH, EC, temperature of water, NH_4_–N, NO_3_–N, NO_2_–N, TKN, TN, PO_4_–P, TP, DO, BOD_5_, COD, TSS, TDS).

The results of the study will help in developing strategies for dealing with water blooms, a reliable system for monitoring pressures causing eutrophication, and optimal technologies for the reconstruction of multifunctional reservoirs, an example of which is the Turawa reservoir. These implications are consistent with the objectives of sustainable development and rational water management strategies in the context of maintaining good quality, defined by acts of national and international laws (e.g. the Water Framework Directive strives to achieve at least good ecological status of surface water bodies by the end of a given planning cycle, currently 2027)^45–47^. The research would contribute to the expansion of knowledge about the mechanisms related to the process of eutrophication, which is a global problem, and respond to the current needs in the field of monitoring the aquatic environment^48^.

## Materials and methods

Water quality tests were conducted on the Turawa reservoir (50°43′25"N, 18°07′13"E), located in south-western Poland (Opolskie Voivodeship). The reservoir was constructed in 1939 along 18.9 km of the Mała Panew River (a right tributary of the Odra River); its catchment area is 1423 km^2^ and consists of 160 elementary catchments^42,43^. It is a multifunctional reservoir (including flood protection, hydropower, recreation, fishing, and shipping); however, due to the intensified eutrophication after the flood in 1997, it does not fulfill its functions. Therefore, it is necessary to conduct water quality analysis and identify sources of pollution to develop a strategy for counteracting water blooms.

**Figure 1:**
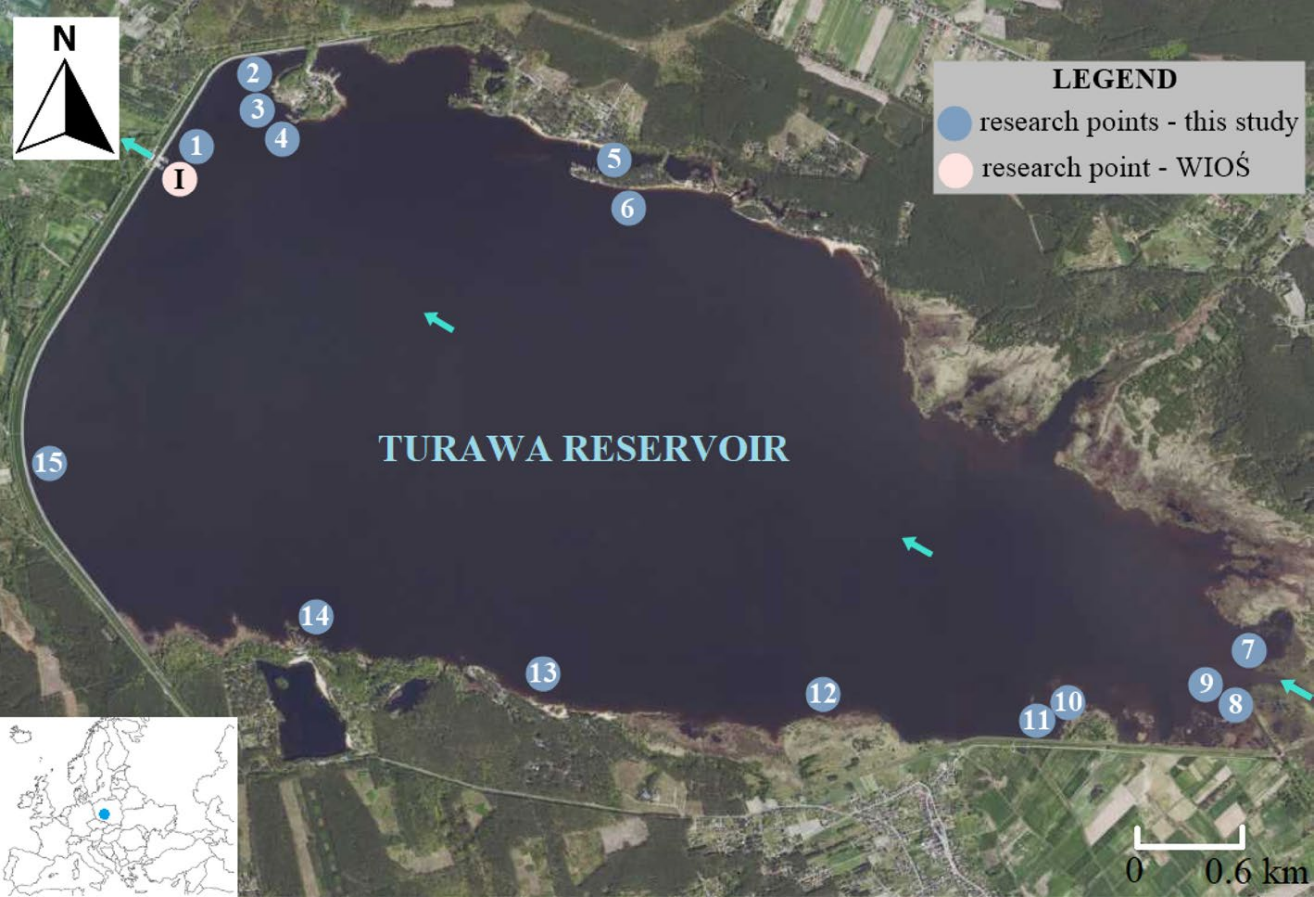
Location of sampling points for physicochemical testing of water in the Turawa water reservoir (south-western Poland; map background – orthophoto, Polish National Geoportal: https://mapy.geoportal.gov.pl/imap/Imgp_2.html?gpmap=gp0).

In the analysis of the water quality of the Turawa reservoir, data from the Province Inspectorate for Environmental Protection (WIOŚ) in Opole^49^ and own research, conducted once a month from January 2019 to December 2020, were used. Water samples for testing were collected from 15 research points in the Turawa reservoir (Fig. 1). The water samples were collected in plastic bottles and transported to the laboratory in a refrigerator. Ex situ laboratory tests were performed at the Environmental Research Laboratory at the Wrocław University of Environmental and Life Sciences. With regard to the WIOŚ studies, the analysis used monthly data from the period 1998–2020 (in 2010, 2012, 2013, 2015, 2017, 2018, measurements were not carried out due to changes in the monitoring system resulting from the implementation of the assumptions of the Water Framework Directive–limiting the number of measurement points within surface water bodies and the scope of analyzes conducted towards only parameters for the purposes of effective management of the surface water quality in the light of the adopted six-year planning cycles), from the measurement and control point "Mała Panew-Turawa reservoir" (surface water body "Mała Panew–Turawa reservoir", 50°44′11.2"N 18°05′30.4"E)^50^, located 100 m away from the dam closing the reservoir (water samples taken from the surface of the reservoir, above the dam, lower part of reservoir; location chosen due to the only research point in the Turawa water reservoir containing long-term observations of water quality).

Table 1 presents the specification of laboratory methods and measuring equipment^51,52^. Parameters such as temperature, pH, and electrical conductivity (EC) were determined in situ using the HI98129 Combo Tester (Hanna Instruments, Woonsocket, RI, USA). The measurement error was from 0.5 to 5.0%, i.e. EC and DO–0.5%, pH and temperature of water–2.0%, other parameters–5.0%^52^.

**Table 1:** Specification of laboratory methods for physicochemical determination of water^51,52^.

No	Parameter	Name of the method	Measurement range
1	pH	Potentiometric method	0.00–14.00
2	Electrical conductivity (EC)	Conductometric method	0.1–2000 mS/cm
3	Temperature of water	Temperature sensor	− 50.0–199.9 °C
4	Ammonium nitrogen (NH_4_–N)	Spectrophotometric method	0.001–1000 mg/L
5	Nitrate nitrogen (NO_3_–N)		0.1–7.0 mg/L
6	Nitrite nitrogen (NO_2_–N)		0.001–1.2 mg/L
7	Total Kjeldahl nitrogen (TKN)	Method after mineralization with Se	–
8	Total nitrogen (TN)	Spectrophotometric method	–
9	Phosphate phosphorus (PO_4_–P)		0.001–0.5 mg/L
10	Total phosphorus (TP)		–
11	Dissolved oxygen (DO)	Electrochemical sensor	0.00–20.00 mg/L
12	Biochemical oxygen demand (BOD_5_)	Dilution method	0.1–2000 mg/L
13	Chemical oxygen demand (COD)	Dichromate method	0.1–1000 mg/L
14	Total suspended solids (TSS)	Filtration though glass-fibre filters	0.01–4000 mg/L
15	Total dissolved solids (TDS)		0–2000 mg/L

The fulfillment of the parametric requirements concerning the state limit values of selected physicochemical parameters was analyzed, and the average ecological status for these parameters was calculated. To make the assessment, the Regulation of the Minister of Infrastructure (June 25, 2021, Poland) on the classification of ecological status, ecological potential, and chemical status, and the method of classifying the status of surface water bodies and environmental quality standards for priority substances, were used. The classification was made for river surface water bodies of surface water type 0, which are dam reservoirs. The full classification for the assessed parameters is presented in Table 2.

**Table 2:** List of limiting values for surface water quality classes of the assessed physicochemical parameters of the Turawa reservoir.

Parameter (unit)	Class I	Class II	Class III	Limit value of the parameter L_P_
pH	6.0–8.5	6.0–9.0	< 6.0, > 9.0	6.0, 9.0
EC (μS/cm)	0–1000	1001–1500	> 1500	1500
TN (mg/l)	0.0–5.0	5.1–10.0	> 10.0	10.0
NO_3_–N (mg/l)	0.0–2.2	2.3–5.0	> 5.0	5.0
PO_4_–P (mg/l)	0.000–0.065	0.066–0.130	> 0.130	0.13
TP (mg/l)	0.00–0.20	0.21–0.40	> 0.40	0.4
DO (mg/l)	≥ 7.0	5.0–6.9	0.0 – 4.9	5.0
BOD_5_ (mg/l)	0.0–3.0	3.1–6.0	> 6.0	6.0
COD (mg/l)	0.0 – 25.0	25.1–30.0	> 30.0	30.0

The assessment of compliance with the standards in relation to the limit values of the analyzed physicochemical parameters (MS_P_) is the quotient of the average concentration of a given indicator from January 2020 to December 2021 (C_P_) to the limit value specified in the above regulation (L_P_), according to formula (1):

1 $$MS_{P} = \frac{{C_{P} }}{{L_{P} }}$$

The final results were classified as follows: 0.00–1.00 points present very good quality, 1.01–2.00 points present good quality, 2.01–3.00 points present moderate quality, 3.01–4.00 points present poor quality, and > 4.00 points present bad quality of the physicochemical parameters.

The average ecological status was determined by assigning a specific number of points to each class of ecological status, i.e., Class I: 1 point, Class II: 2 points, and Class III: 3 points. Then, the average of these results was calculated for each parameter and pointed on the following scale: Class I: 1.00–1.66 points; Class II: 1.67–2.33 points; and Class III: 2.34–3.00 points.

The above information will assist in analysing the contribution of parametersin the water quality deterioration of the Turawa reservoir (division of results into physicochemical parameters) and locating the occurrence of deteriorations (division of results into research points). This will further allow accurate identification of sources and locations of water pollution leading to eutrophication.

The determination of three water quality indices (National Sanitation Foundation water quality index, NSF WQI; universal water quality index, UWQI; and Oregon water quality index, OWQI) and trophic status indices (Vollenweider, Carlson, TLI) on the basis of WIOŚ data from the measurement point located in the Turawa reservoir, between the period 1998–2020^49^, i.e.:

- The NSF WQI, modified by the Scottish Development Department^53^ and others^54–56^, included 7 physicochemical parameters. This method uses the following formula, where the parameters are valued on a scale of 0 to 100, and the ratio of the number of substandard results (according to Tiable 1) to the total number of results (q_i_) and the weight for each of them (w_i_), i.e., DO = 0.24, BOD_5_ = 0.20, COD = 0.15, TP = 0.17, EC = 0.11, NO_3_–N = 0.09, and pH = 0.04, is calculated:
  2 $$NSF\;WQI = \frac{1}{100}\left[ {\mathop \sum \limits_{i = 1}^{n} q_{i} \cdot w_{i} } \right]^{2}$$

The following classification was adopted: 0.00–11.10, 11.11–33.30, 33.31–55.60, 55.61–83.30, and 83.31–100 points indicate very good, good, reasonable, polluted, and very polluted water qualities, respectively.

- UWQI is calculated from the results for 5 physicochemical parameters (BOD_5_, NO_3_, DO, TP, and pH) of the considered multi-year period. The 90th or 10th percentile was included, depending on the parameter (NO_3_, TP, BOD_5_, and pH for 90th percentile; DO and pH for 10th percentile; thus, possible measurement errors were taken into account; for pH, two percentiles were considered due to the optimum parameter being in the middle of the scale—both too low and too high values will indicate poor water quality, and measurement errors may occur at both ends of the scale). Then, subindices are calculated for each parameter (values from 0 to 100) using the formulas presented in the literature^57–59^. Lastly, the weighted average for the sub-indices calculated in this way is determined by multiplying their values by the weights assigned to them, i.e., respectively: BOD_5_, 0.166; NO_3_, 0.251; DO, 0.332; TP, 0.166; and pH, 0.085. The final rating for this method is as follows: 0.0–24.9, poor water quality; 25.0–49.9, marginal water quality, 50.0–74.9, fair water quality, 75.0–94.9, good water quality, and 95.0–100.0, excellent water quality.
- OWQI is calculating by taking medians for 6 parameters (in this case: DO, BOD_5_, NH_4_ + NO_3_–N, TP, pH, and temperature of water). The sub-index values from appropriate formulas for each of the parameters are calculated^58–60^, and lastly, the arithmetic mean of the results obtained in this way is determined and converted into a point scale expressed from 10 to 100 into a scale from 0 to 100 points, as follows:
  3 $$OQWI = 1.111 \times OQWI_{raw} - 11.11$$

where OWQI_raw_ is calculated water quality index, expressed on a scale of 10 to 100. The classification for this method is as follows: 0–55.00 points present very poor; 55.01–77.75 points present poor; 77.76–83.25 points present fair; 83.26–88.75 points present good; and 88.76–100 points present excellent water quality (Table 3).

- Carlson index (TSI_P_)^61^ determines the trophic status and is calculated from the formula (4) and assessed according to four classifications listed in Table 3, where C_P_ is average annual concentration of phosphorus [g/l]:
  4 $$TSI_{P} = 14.42\;{\text{l}} \;C_{P} + 4.15$$
- The methods of Vollenweider^63^ and Kajak^64^, determine the degree of eutrophication risk in a water reservoir from total phosphorus (TE_P_) and total nitrogen (TE_N_), determined by the following formulas (5–6):
  5 $$TE_{P} = \frac{{DL_{P} }}{{AL_{P} }}$$
  6 $$TE_{N} = \frac{{DL_{N} }}{{AL_{N} }}$$

**Table 3:** Classification of the trophic status of reservoirs based on the Carlson index due to the phosphorus load (TSI_P_)^61,62^.

Reference@Trophic status	Vollenweider (1965)	Sakamoto (1966)	EPA survey (1974)	Carlson and Simpson (1996)
Oligotrophic	< 10	< 20	< 10	< 6
Oligo-mesotrophic	10–20	–	–	6–12
Mesotrophic	20–50	20–50	10–20	12–24
Mesoeutrophic	50–100	–	–	24–48
Eutrophic	> 100	> 50	> 20	48–96
Hipereutrophic	–	–	–	> 96

where DL_P_ is dangerous load of phosphorus [g/m^2^ year]; AL_P_ is actual phosphorus load [g/m^2^ year] (AL_P_ = $\frac{{C_{P} \cdot Q_{mean} }}{A}$; where Q_mean_ is average annual flow [l/year], and A is reservoir area [m^2^]); DL_N_ is dangerous load of nitrogen [g/m^2^ year] (the value of 2.0 was assumed, according to the Kajak method (2001)); and AL_N_ is actual nitrogen load [g/m^2^ year] (AL_N_ = $\frac{{C_{N} \cdot Q_{mean} }}{A}$; where C_N_ is average annual nitrogen concentration [g/l]).

- Trophic Level Index (TLI)^65–67^ is calculated using formulas (7–11) that use the average results for the following parameters: total nitrogen (TN) (TL_N_ index), total phosphorus (TP) (TL_P_ index), water transparency measured with a Secchi disc (TS) (TL_S_ index), and chlorophyll a (TC) (TL_C_ index). The overall score is expressed on a scale of 0 to 9 and is assessed based on the scale shown in Table 4:
  7 $$TLI = 0.25 \, \left( {TLn + TLp + TLs + Tlc} \right)$$
  8 $$TL_{N} = - 3.61 + 3.01\;\log \, \left( {TN} \right)$$
  9 $$TL_{P} = 0.218 + 2.92\;\log \;\left( {TP} \right)$$
  10 $$TL_{S} = 5.10 + 2.27 \, \;\log \;\left( {1/SD{-}1/40} \right)$$
  11 $$TL_{C} = 2.22 + 2.54\;\log \left( {Chl} \right)$$

**Table 4:** Classification of trophic status using the Trophic level index (TLI)^65–67^.

TLI value	Trophic status	Itemization
0.0–2.0	Microtrophic	Very clean water, with very low levels of nutrients and algae, often glacial water, very good water quality
2.01–3.0	Oligotrophic	Low levels of nutrients and algae, clear and blue water, good quality
3.01–4.0	Mesotrophic	Medium levels of nutrients and algae, moderate quality
4.01–5.0	Eutrophic	High amounts of nutrients and algae, cloudy water, poor quality
5.01–9.0	Supertrophic	Very high amounts of phosphorus and nitrogen, significant algae blooms, poor water clarity, usually not meeting standards for recreation, very poor quality

Statistical analyses of the data collected in the period 2019–2020 were also performed, enabling a more complete interpretation of the results (for *p* < 0.05), i.e.:

- Basic descriptive statistics for the analyzed parameters, i.e., minimum, maximum, mean, median, and standard deviation.
- Non-parametric Mann–Whitney U test^68^, in which the results for each physicochemical parameter were compared in two data groups: 2019 and 2020. A null hypothesis that the medians in both groups of variables are equal and their distribution is different than normal, was assumed in this study. The values of U and z-ratio were calculated according to the following formulas (12–13):
  12 $$u = \frac{{n\left( {n + 1} \right)}}{2} - \sum_{ranks}$$
  13 $$Z = \frac{{U - \sigma_{U} }}{{x_{U} }}$$

where n is the number of items in the samples, ∑_ranks_ is the sum of ranks in the sample, σ_U_ is the standard deviation of U, and x_U_ is the mean of U.

- HCA shows the potential grouping of the assessed parameters and clusters them into specific groups based on similarities, such as the scale of anthropogenic pressures, origin of pollutants, and their locations, the cluster method was group average and distance type was Euclidean method^69^.
- A correlation matrix using Spearman's rank correlation coefficients for non-linear variables (non-parametric measure of monotonic relationship between random variables) shows the relationships between all considered physicochemical parameters. This measure allows determination of the direction and strength of correlation, where R >|0.7| means strong, R =|0.5–0.7| means moderate, R =|0.3–0.5| means weak, and R <|0.3| means very weak or absent correlation^70^.
- PCA can identify the initial variables that have a large impact on the appearance of individual principal components (in this case, the variability within the evaluated parameters or points). It also serves to reduce dimensions in the analyzed data set^71^. To perform this analysis properly, the following steps were performed^72^:
- Preliminary analysis, showing the size of variance in each variable and enabling the selection of variables for PCA:
- The Bartlett's sphericity test—the null hypothesis is tested that the correlation matrix is an identity matrix, i.e. the variables are orthogonal, uncorrelated; if the variables are perfectly correlated, then r = 1, if there is no correlation, then r = 0; *r* values < 0.5 are discarded; PCA can be performed if the test shows statistical significance (*p* < 0.05);
- Communalities analysis – the proportion of variance of each variable that can be explained by factors (the sum of the squared loadings for variables); ranges from 0 to 1, where 0 means no match to the factor solution and 1 means a perfect match; values below 0.5 are rejected due to poor representation of the isolated components (after extraction).
- Proper analysis—after selection of variables for PCA:
  - Re-run the preliminary analysis to verify that it met its assumptions after discarding unmatched variables;
  - After a positive analysis, extracting components with eigenvalues greater than 1.0 on the basis of a scree plot (these components are responsible for the greatest extent for the variability of variable variances); the remaining components are discarded from the analysis;
  - Analysis within the components based on the explanation of the total variance (determining how many % of cases are explained by a given component) and the assignment of individual variables (parameters, points) to a given component based on the component matrix (the higher the value, the greater the correlation with the given component);
  - Interpretation of the data presented graphically in the PCA chart for the two components with the highest eigenvalue (explaining the largest percentage of variables). The analysis is performed by checking the distance between the individual variables in space, as well as by referring to their position relative to the X and Y axes (i.e. principal components 1 and 2 – PC1 and PC2, respectively).

Softwares, SPSS Statistics 26 (IBM, Armonk, NY, USA), Origin Pro 2021b and 2022b (OriginLab Corporation, Northampton, MA, USA), Statistica 13.3 (StatSoft Polska, StatSoft, Inc., Tulsa, OK, USA), Microsoft Office 2013 and 2021 (Microsoft, Richmond, WA, USA), were used for statistical analyses and drawings. Maps were created based on the results of our own research in ArcGIS 10.5.1 (Esri Inc., Redlands, CA, USA), the map background (outline of the Turawa reservoir) was the 1:10,000 Map of the Hydrographic Division of Poland, and the interpolation was performed using the Inverse Distance Weighted (IDW) method.

## Results

### Descriptive statistics

Table 5 presents descriptive statistics for the test results of physicochemical parameters from points located in the Turawa reservoir in 2019–2020. Considering the differences in the median between years (dividing the 2020 median or mean by the 2019 value and subtracting 1; values greater than 0% mean that they were higher in 2020 than in 2019, and less than 0% – lower), the highest values were recorded for NO_3_–N (807.14%), DO (726.92%), TP (642.86%), and PO_4_–P (450.00%), and the largest differences between the means were obtained for NO_3_–N (336.85%), PO_4_–P (266.53%), NH_4_–N (− 98.21%), and DO (97.59%). The Mann–Whitney U test indicates that for 11 out of 14 physicochemical parameters, statistical significance was obtained (except for TN, TSS, and pH).

**Table 5:** Descriptive statistics for the results of physicochemical parameters of the Turawa water reservoir in 2019 –2020.

Parameter	Year	Statistics							U Mann–Whitney test		
		Min	Max	Median	Mean	SD	∆_Median20/19_ (%)	∆_Mean20/19_ (%)	U	z	p
pH	2019	5.6	9.4	8.2	7.92	0.88	− 3.66	0.21	9878	1.58244	0.1141
	2020	6.7	9.5	7.9	7.94	0.58					
EC	2019	236	509	339.5	348.15	55.88	15.76	12.13	5290.5	− 7.75282	< 0.00001
	2020	233	543	393	390.40	51.10					
TN	2019	0.55	21.69	3.11	4.48	3.73	13.67	− 4.21	7164	1.28425	0.20054
	2020	1.02	13.77	3.535	4.29	2.56					
TKN	2019	0.55	21.65	2.105	3.64	3.85	− 17.81	− 37.70	6628.5	− 2.21171	0.0271
	2020	0.78	12.27	1.73	2.26	1.75					
NH_4_–N	2019	0.01	533.25	0.67	14.45	64.74	− 71.64	− 98.21	5734	− 4.38311	< 0.00001
	2020	0	1.71	0.19	0.26	0.26					
NO_3_–N	2019	0	4.16	0.14	0.46	0.71	807.14	336.85	3588.5	− 10.04208	< 0.00001
	2020	0.03	10.65	1.27	2.02	2.12					
NO_2_–N	2019	0	3.7	0.05	0.34	0.74	− 40.00	− 86.10	7328.5	5.01162	< 0.00001
	2020	0.01	0.8	0.03	0.05	0.09					
PO_4_–P	2019	0	0.33	0.01	0.018	0.032	450.00	266.53	3881.5	− 8.69181	< 0.00001
	2020	0	1.01	0.055	0.064	0.098					
TP	2019	0	81.31	0.07	1.58	7.99	642.86	− 60.18	6631	− 5.94979	< 0.00001
	2020	0.06	5.54	0.52	0.63	0.66					
DO	2019	0	39.68	1.04	4.39	5.94	726.92	97.59	4863.5	− 8.23715	< 0.00001
	2020	0.3	14	8.6	8.67	2.46					
BOD_5_	2019	0.24	348	2.8	13.44	53.53	21.43	− 50.51	4558.5	2.73468	0.00634
	2020	0.4	131.6	3.4	6.65	14.23					
COD	2019	0	904	9.25	26.66	105.42	22.16	− 27.54	8135.5	− 3.92618	0.00008
	2020	1.9	398.4	11.3	19.31	41.59					
TSS	2019	5	12,720	45	321.04	1340.08	11.11	− 80.57	9646.5	0.12964	0.89656
	2020	3	1080	50	62.39	101.59					
TDS	2019	170	400	260	263.45	43.98	− 18.85	− 19.88	4038.5	8.39109	< 0.00001

Furthermore, for spatio-temporal analyses, parameters with convergent positive or negative differences in the median and mean for the analyzed years compared to the previous year, were selected (NO_3_–N, DO, PO_4_–P, NH_4_–N, NO_2_–N, TDS, TKN, and EC were selected, and TP, COD, BOD_5_, TN, and TSS were discarded) and at the same time statistical significance was obtained for them (NO_3_–N, DO, TP, PO_4_–P, NH_4_–N, NO_2_–N, COD, BOD_5_, TDS, TKN, EC; TN, TSS, and pH were rejected). After combining both criteria, 8 out of 14 parameters were selected for analysis, i.e., NO_3_–N, NO_2_–N, NH_4_–N, TKN, PO_4_–P, DO, TDS, and EC.

### Temporal and spatial variability of physicochemical parameters of water

The highest average annual concentration values in 2019–2020 were recorded for most parameters in July and August, corresponding to the highest intensity of water blooms in the Turawa reservoir. The lowest values of the tested parameters were observed in November and December, when the algae vegetation was significantly extinguished due to the prevailing low temperatures and worse light conditions than in the summer months.

**Figure 2:**
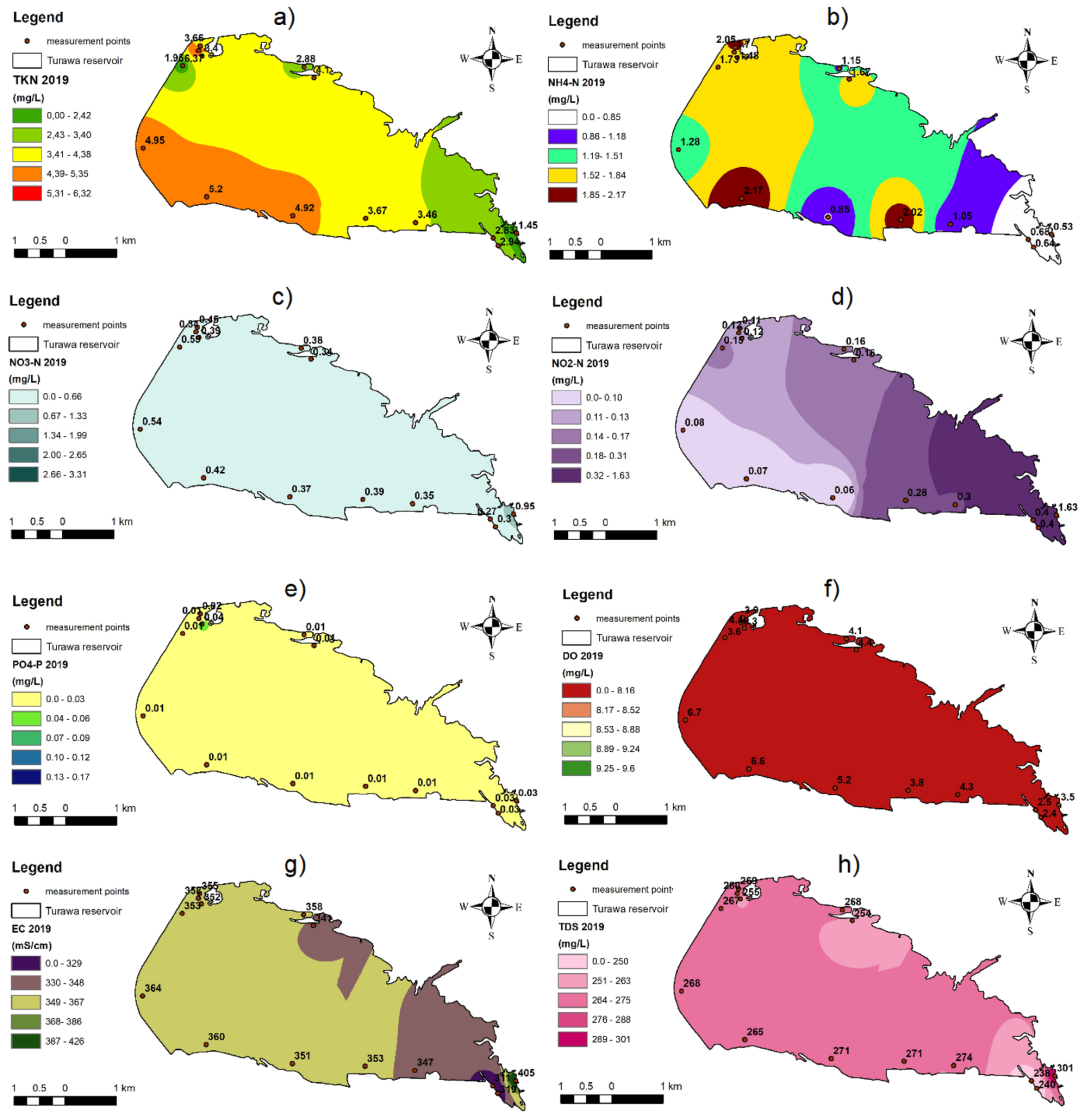
Maps of spatial distribution of selected physicochemical parameters on the Turawa reservoir in 2019: (**a**) TKN, (**b**) NH_4_–N, (**c**) NO_3_–N, (**d**) NO_2_–N, (**e**) PO_4_–P, (**f**) DO, (**g**) EC, and (**h**) TDS (maps generated in ArcGIS 10.5.1, background map: the 1:10,000 Map of the Hydrographic Division of Poland, interpolation: Inverse Distance Weighted method).

In 2019 (Fig. 2), the noticeably lowest values of the tested parameters (especially nutrients) were present at points on the reservoir that were remote from human settlements (Points 10, 11, and 12), and the highest were at human agglomerations that were a source of organic matter (mainly hotels, holiday homes, and resorts near Points 3, 7, 13, 14, and 15). Additionally, the impact of pollutants flowing in from the Mała Panew River in the south-eastern part of the Turawa water reservoir was noticeable (Points 8 and 9), as can be seen in the values of the following parameters: NH_4_–N, NO_3_–N, NO_2_–N, PO_4_–P, DO, EC, and TDS.

**Figure 3:**
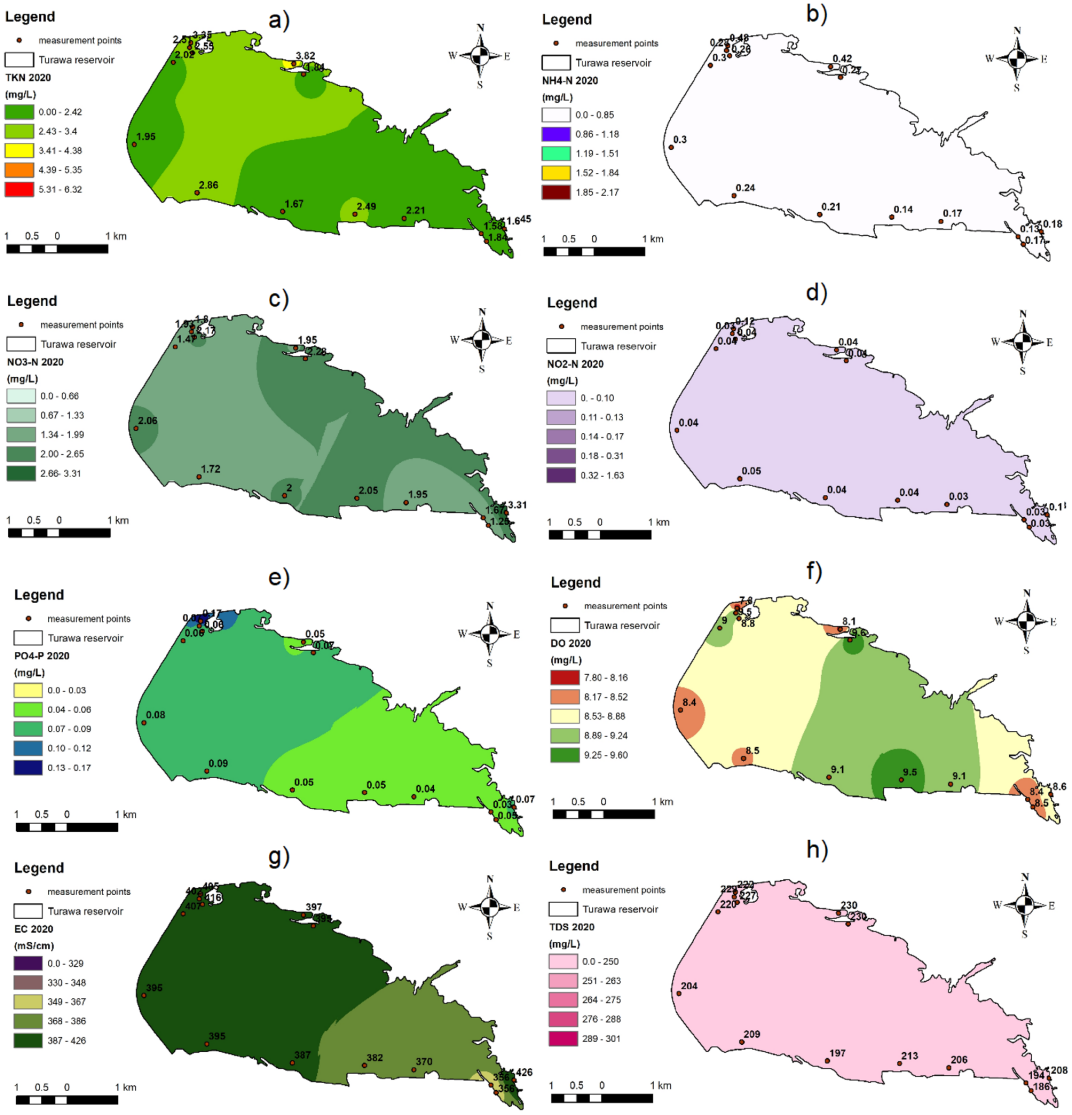
Maps of spatial distribution of selected physicochemical parameters on the Turawa reservoir in 2020: (**a**) TKN, (**b**) NH_4_–N, (**c**) NO_3_–N, (**d**) NO_2_–N, (**e**) PO_4_–P, (**f**) DO, (**g**) EC, and (**h**) TDS (maps generated in ArcGIS 10.5.1, background map: the 1:10,000 Map of the Hydrographic Division of Poland, interpolation: Inverse Distance Weighted method).

The average annual concentrations of parameters for the Turawa reservoir in 2020 (Fig. 3) indicate a large impact of anthropogenic pressures, i.e., nearby holiday resorts, especially in the northern part of the reservoir (Points 3 and 5). This can be seen mainly in relation to biogenic parameters, i.e., TKN, NH_4_–N, NO_2_–N, and PO_4_–P. Additionally, in the case of DO, EC, NO_3_–N, and NO_2_–N, the impact of pollutants carried by the waters of the Mała Panew River was noticeable (similar to 2019; Points 8 and 9), flowing into the reservoir from the south-east (near the Jedlice pumping station and preliminary reservoir), where more unfavorable values of these parameters were recorded (lower for DO, higher for EC, NO_3_–N, and NO_2_–N).

Among the 8 physicochemical parameters considered, the largest absolute differences in average annual values at all points in the Turawa water reservoir between 2020 and 2019 were obtained, decreasingly, for: NO_3_–N (336.85%), PO_4_–P (266.53%), NH_4_–N (− 98.21%), DO (97.59%), NO_2_–N (− 86.10%), TKN (− 37.70%), TDS (− 19.88%), and EC (12.13%).

### Ecological status

#### Averaged ecological status

**Figure 4:**
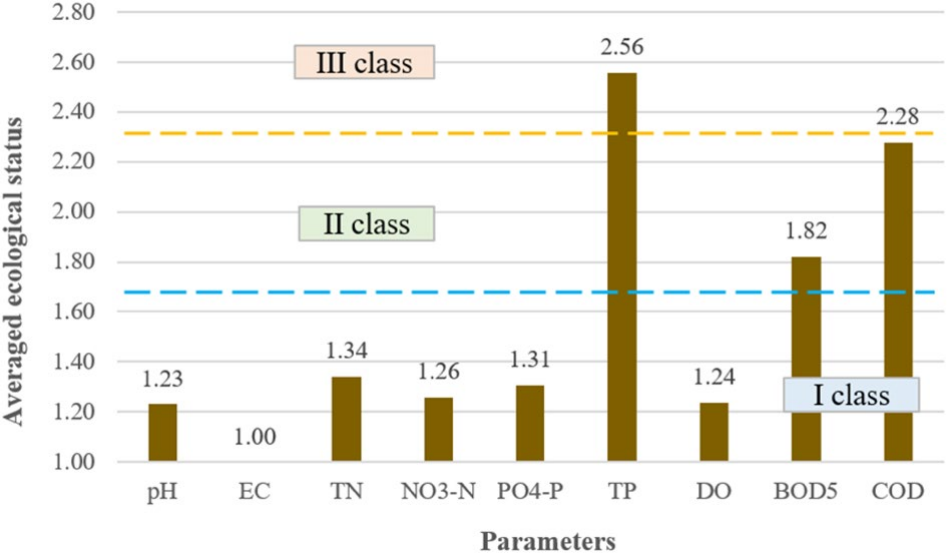
Averaged ecological status by parameters.

The averaged ecological status indicates that the parameters responsible for the greatest depletion of the water quality in the Turawa reservoir were TP (2.56), COD (2.28), and BOD_5_ (1.82); here TP belonged to class III water quality and was below good condition, while BOD_5_ and COD were class II and had good status. In other cases, the values of the calculated indicator ranged from 1.00 (EC) to 1.31 (PO_4_–P), i.e., class I of the ecological status of the parameters (very good condition). Figure 4 presents the described results of the averaged ecological status for the parameters.

**Figure 5:**
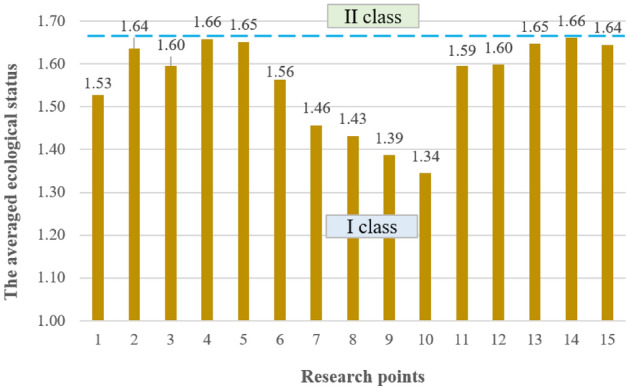
Averaged ecological status by points.

For points, a large variability was not observed in values from 1.34 (Point 15) to 1.66 (Points 5 and 19). These values qualified all assessed points for class I of ecological status, i.e., very good status (as shown in Fig. 5). Therefore, on a general, averaged scale, it was impossible to indicate points that could have clearly worsened the quality of water in the Turawa reservoir.

#### Indicators of exceeding ecological state limit values

**Figure 6:**
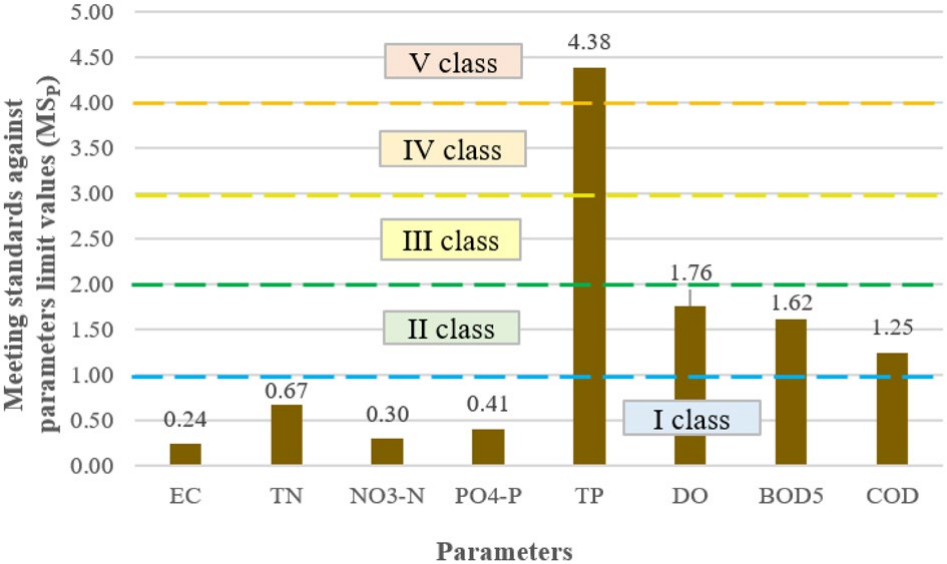
Indicators of exceeding ecological status limit values by parameters.

As shown in Fig. 6, high concentrations of total phosphorus, classified as a very poor quality parameter, were responsible for the deterioration of water quality in the Turawa water reservoir. Limiting values were also exceeded for oxygen parameters, i.e., DO, BOD_5_, and COD, but they were not as high as for TP. All these factors are the cause of eutrophication of reservoirs, especially phosphorus compounds, which are used by algae participating in the processes of water blooms to rapidly grow their biomass. The above results are consistent with the calculated values of the average ecological status.

**Figure 7:**
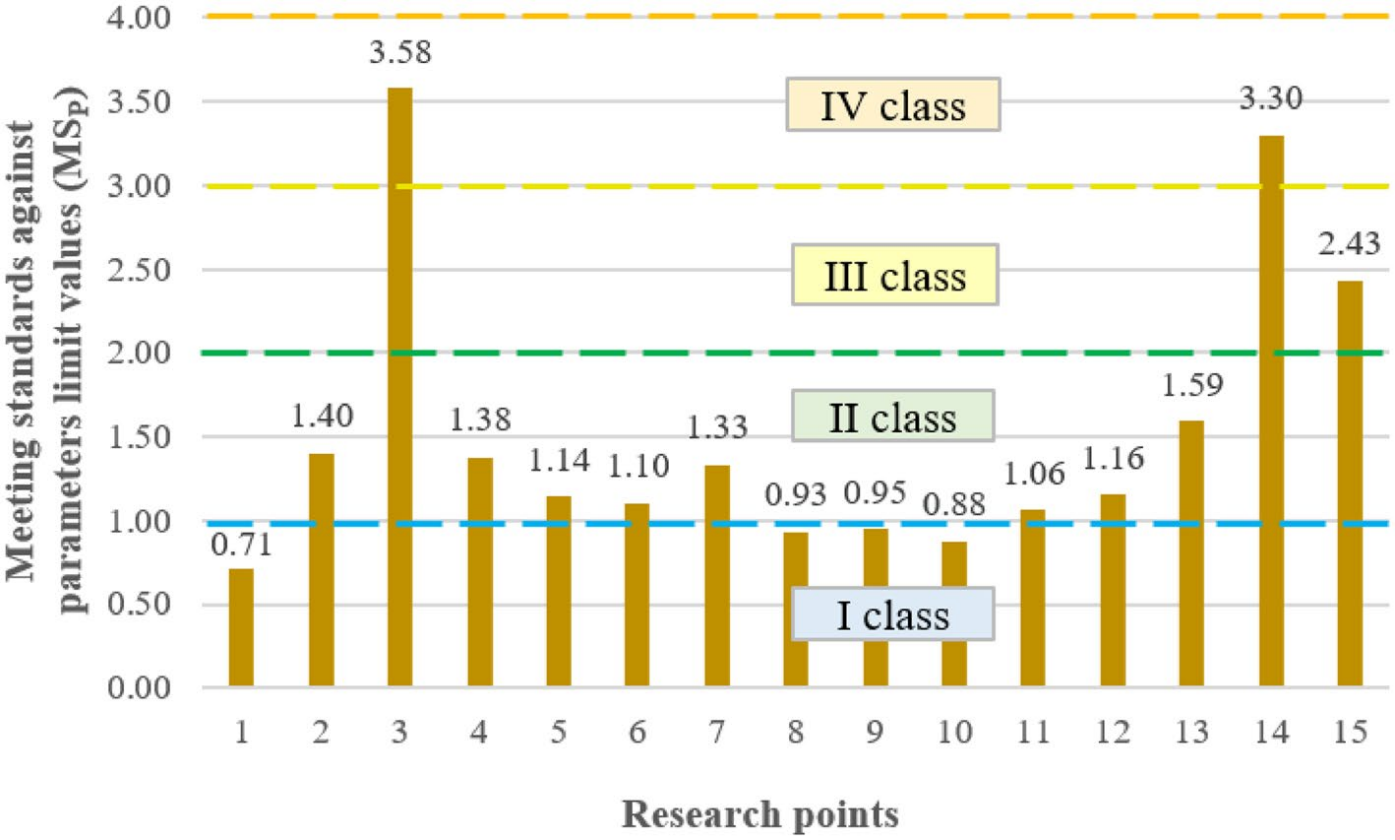
Indicators of exceeding ecological status limit values by points.

The sources of pollution in 2019–2020 were mostly control Points 3 and 14 (poor water quality) and Point 15 (moderate water quality). In the remaining cases, the water quality was classified as very good or good (only in 4 out of 15 points exceeded the limiting values of parameters). Points 3 and 14 are located in places with high tourist intensity, near local holiday centers and bathing areas, and Point 15 is a place intensively used for fishing. Figure 7 shows the indicators of the ecological status limit values by points.

The results of the quality standards confirm that the eutrophication occurring in the reservoir was caused by the activity of blue-green algae and had the greatest impact on the deterioration of water quality. In most of the examined months, the exceedances concerned TP, DO, COD, and BOD_5_. TP is responsible for the greatest extent of water blooms, as it is the basic source of food for organisms involved in this process, while DO, BOD_5_, and COD indicate a significant deterioration of oxygen conditions due to the fact that algae largely use this oxygen to carry out their life processes. The phenomena described above intensify in the summer months (July–September), when higher water temperatures are conducive to the development of phytoplankton and result in the greatest increase in biomass. The general conclusion from the analyses is that water blooms, combined with the supply of nutrients and deteriorating aerobic conditions for the life of aquatic organisms, are the biggest problems in the Turawa reservoir that need to be resolved.

### Water quality

#### The National sanitation foundation water quality index (NSF WQI)

**Figure 8:**
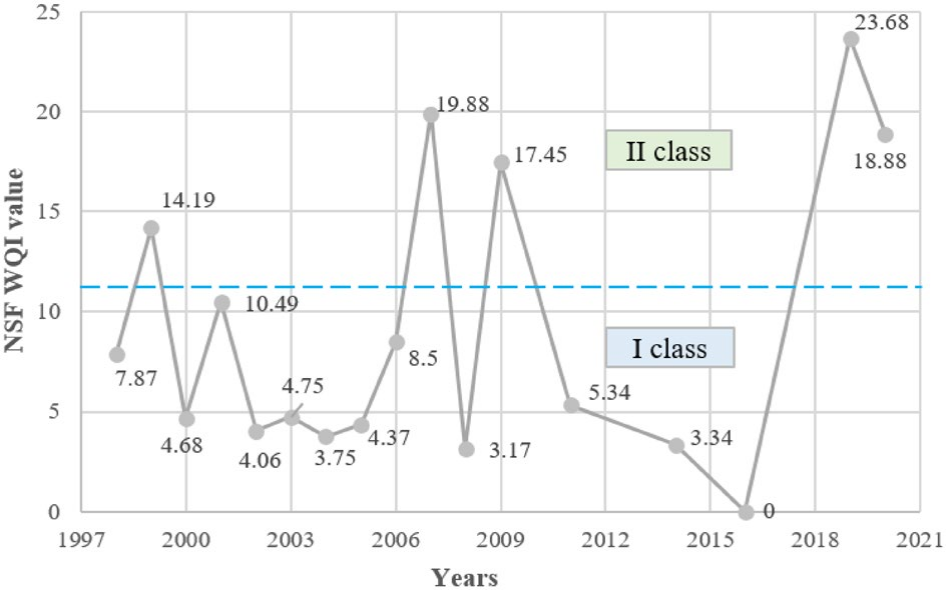
Variability in National Sanitation Foundation water quality index of the Turawa reservoir in 1998–2020.

The calculated NSF WQI values (Fig. 8) indicate that the water quality of the Turawa reservoir in 1997–2020 was in classes I or II of water quality (very good or good water quality), in the range of values from 0 (2016) to 23.68 points (2019). Class II was recorded in 1998, 2001, 2007, 2009, 2019, and 2020. The deterioration of the calculated index was mainly due to COD and, to a lesser extent, TP, BOD_5_, and DO, i.e., parameters characterizing the aerobic conditions of water and enrichment in the main nutrient causing eutrophication.

#### Universal water quality index (UWQI)

**Figure 9:**
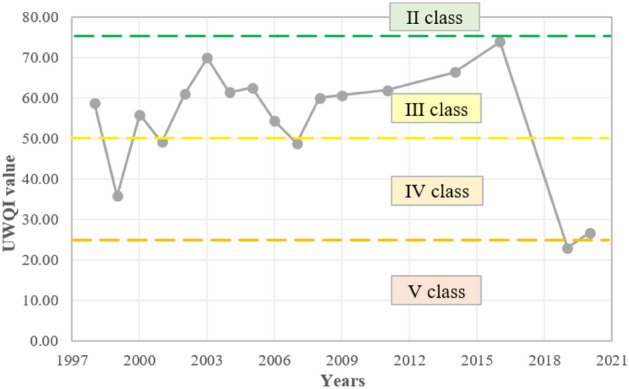
Variation in the universal water quality index of the Turawa reservoir in 1998–2020.

According to the UWQI, most of the test results indicate class III water quality (fair water quality; Fig. 9). The exceptions were the years 2019 (water quality class V, poor water quality), and 1999, 2001, and 2020 (water quality class IV, marginal water quality). The index ranged from 22.94 to 73.81 points in period from 1998 to 2020. The deterioration of the final values of the index (excluding the weights for the parameters) was mainly caused by high concentrations of TP and NO_3_ and too wide fluctuations in pH during a year (averages of the sub-indices: 34.94, 35.24, and 44.12, respectively, on a scale from 0 to 100 points), which indicates enrichment of water with nutrients and disturbances in pH values, e.g., by the activity of aquatic organisms that changes the chemical composition in water. It is worth noting that a drastic deterioration in the index value was noticeable, especially when compared over the years 2016–2019 (3.22 times).

#### Oregon water quality index (OWQI)

**Figure 10:**
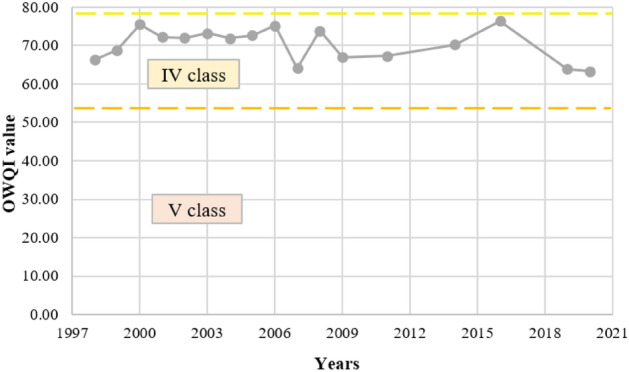
Variation of the Oregon water quality index on the Turawa reservoir in 1998–2020.

All results of the calculated OWQI indicate class IV of water quality (poor water quality) and ranged from 63.35 (in 2020) to 76.39 (in 2015) (Fig. 10). The maximum difference between the index values was, therefore, equal to 20.58%. The deterioration of the final index value was mainly due to high medians of NH_4_ + NO_3_-N (37.68) values and, to a lesser extent, TP (52.76) and BOD_5_ (59.30) (average values of sub-indices for many years, expressed on a scale from 0 to 100 points).

#### Comparison of water quality index results

**Figure 11:**
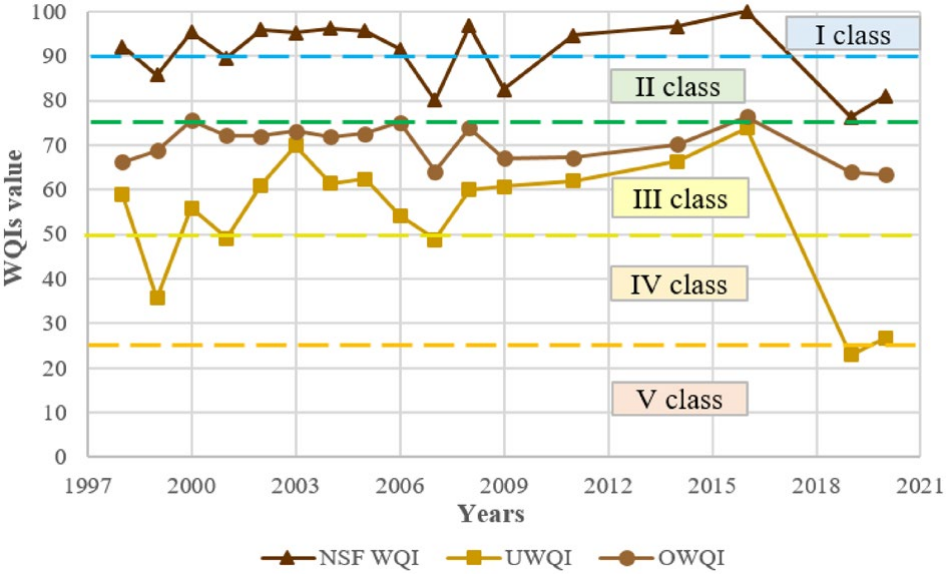
Comparison of water quality indices of the Turawa reservoir in 1998–2020.

Considering aggregate results (Fig. 11), the average water quality was in class II or III (i.e., good or moderate water quality; values from 54.40 in 2019 to 83.40 in 2016). In the case of the NSF WQI, in 6 out of 17 analyzed years, the water quality was good (class II), and in the remaining 11 years, it was very good (class I). For OWQI, these values were already lower, characteristic of classes II or III. The weakest values were recorded for UWQI, i.e., within the III, IV, or V water quality classes. There is a noticeable deterioration in water quality for each water quality index for the years 2016–2019/2020. The most common parameters responsible for lower values of water quality indices were TP, NO_3_ (NO_3_–N), and BOD_5_.

### Trophic status

#### Carlson index (TSI_P_)

**Figure 12:**
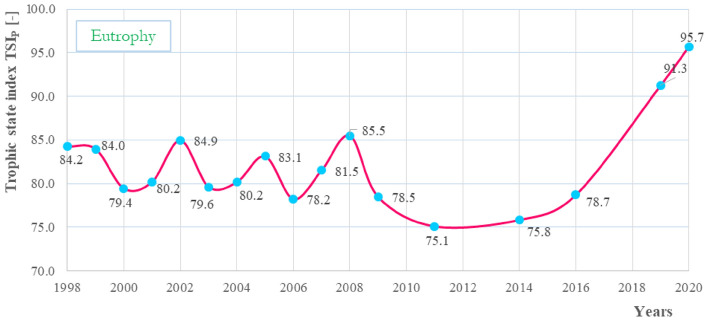
Carlson index of the Turawa reservoir in 1998–2020.

Studies on the trophic status of phosphorus concentration indicate that in each of the Carlson index classifications, Turawa reservoir should be classified as water strongly enriched in this nutrient. Carlson index values ranged from 75.1 in 2011 to 95.7 in 2020 (eutrophic to mesoeutrophic status) and showed an increasing trend, as shown in Fig. 12. Eutrophy of a reservoir means a strong tendency for algal blooms with serious consequences on the environment, society, and economy, which are described in detail in the “Discussion” Section.

#### Vollenweider and Kajak criteria

**Figure 13:**
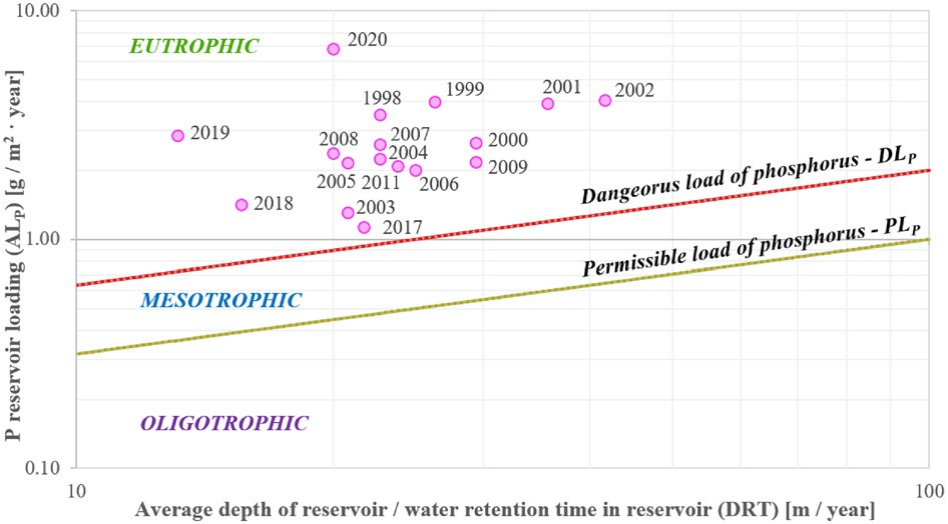
Dependence of permissible and dangerous phosphorus loads for a 5 m deep reservoir together with the results of total phosphorus measurements for the Turawa reservoir.

**Figure 14:**
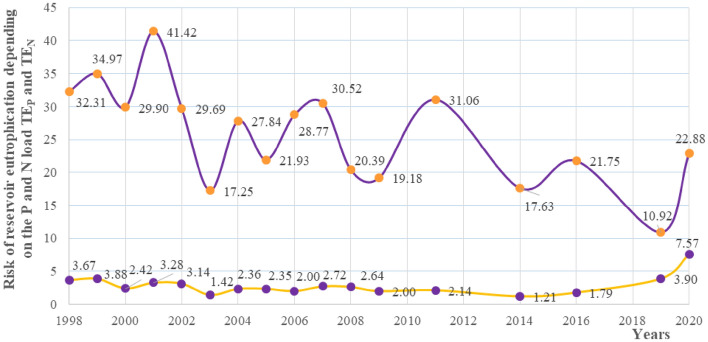
Calculated risk factors for eutrophication of the Turawa water reservoir due to the load of phosphorus (TE_P_) and nitrogen (TE_N_).

Using the Vollenweider criterion, considering the average retention time of the Turawa reservoir and the theoretical curves determining the acceptable and dangerous content of the phosphorus load, the water of the Turawa reservoir was classified as eutrophic (Fig. 13). Furthermore, in 1998–2020, dangerous levels of phosphorus and total nitrogen exceeded the Vollenweider and Kajak criteria, respectively. In the case of phosphorus (TE_P_), the level changed from 1.42 in 2003 to 7.57 in 2020, and nitrogen (TE_N_) from 10.92 in 2019 to 41.42 in 2001 (Fig. 14). The average exceedances were 2.85 and 25.79 in terms of dangerous loads of phosphorus and nitrogen, respectively. In this method, the greater risk of eutrophication was a result of the high load of nitrogen. Simultaneously, it should be noted that the Kajak criterion is more general, so the final errors in the results will be larger than in the Vollenweider method, in which the acceptable and dangerous levels of phosphorus depend on the ratio of the average depth of the reservoir to the retention time in a given year, and are not set top-down for the entire range of parameters, as in the case of the Kayak criterion for nitrogen (in this case, a dangerous nitrogen load of 2 g/m^2^ per year). Summarizing the exceedance values for both nutrients, the highest risk of eutrophication was recorded in 2001 (44.70), 1999 (38.85), and 1998 (35.98), and the lowest in 2019 (14.82), 2003 (18.67), and 2014 (18.84). Considering the TEN:TEP ratio, the largest values (i.e., the relatively largest impact of the TN charge in relation to the TP charge) were obtained for the years 2011 (14.55), 2014 (14.53), and 2006 (14.47), the smallest in 2019 (2.80) and 2020 (3.02), and the mean value was 10.32.

#### Trophic level index (TLI)

**Figure 15:**
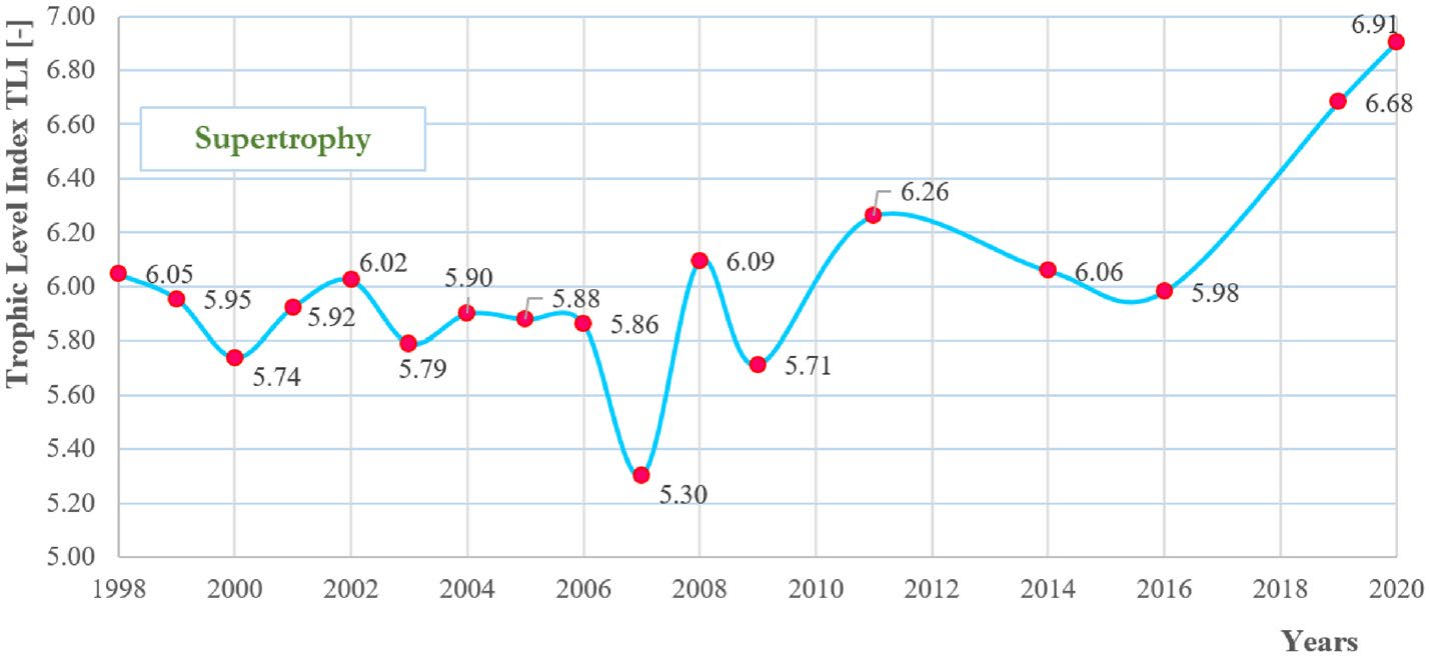
Trophic Level Index of the Turawa reservoir in 1998–2020.

The TLI values calculated for 1998–2020 (Fig. 15) indicate that the trophic status of the Turawa water reservoir had the characteristic of supertrophy throughout the study period (very high amounts of phosphorus and nitrogen, significant algae blooms, poor water transparency, usually not meeting the standards for recreation, and very poor water quality). TLI ranged from 5.30 in 2007 to 6.91 in 2020, averaged at 6.01. The parameters deteriorating the quality of this index were in descending order of mean values at TN (7.36), TP (7.08), and chlorophyll a (6.11). Water transparency was moderate and had characteristics of mesotrophy (values usually ranges from 3.0 to 4.0).

### Multivariate statistics

#### Hierarchical cluster analysis (HCA)

**Figure 16:**
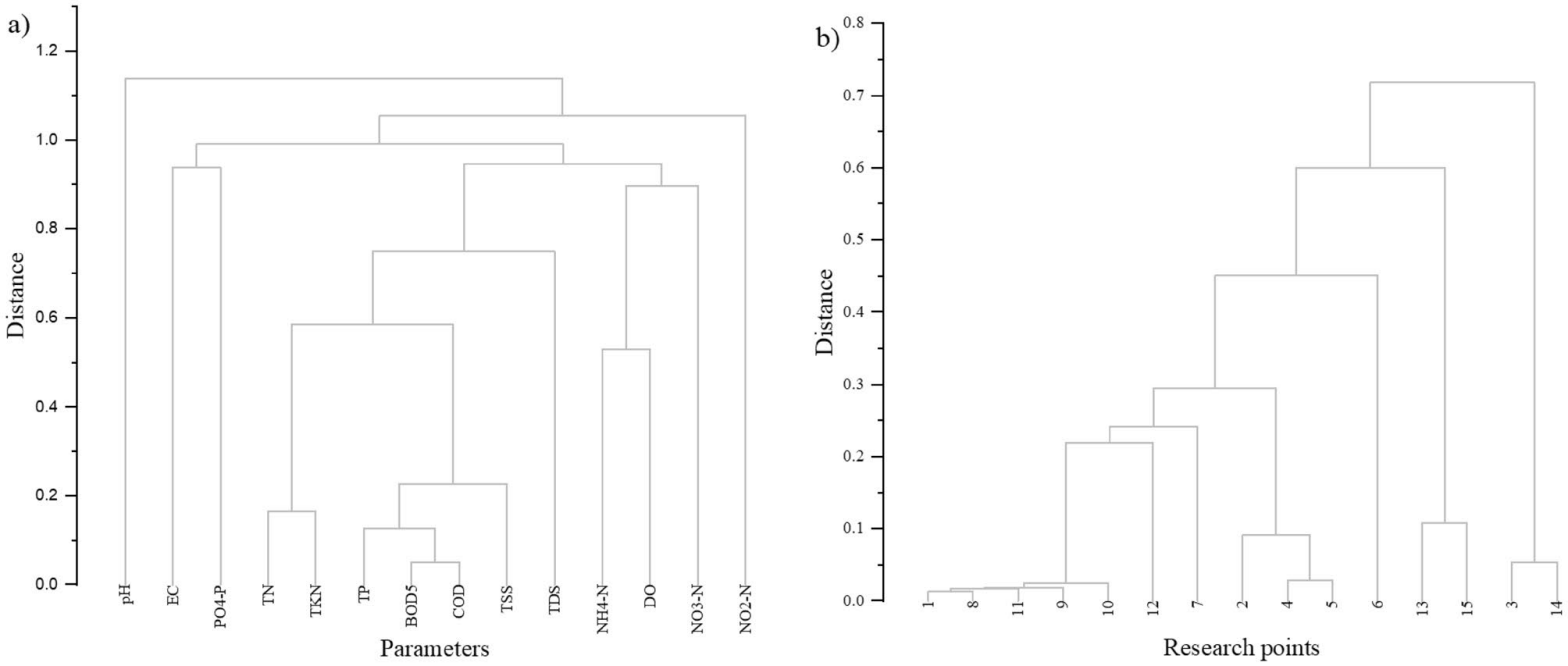
Hierarchical Cluster Analysis (HCA) for the results of (**a**) physicochemical parameters of water and (**b**) research points of the Turawa reservoir in 2019–2020.

The HCA performed for on the water quality parameters (Fig. 16) indicates that the results for pH significantly differed from other parameters at the assessed research points on the Turawa reservoir in 2019–2020 and can be distinguished as a separate data group. In the second group, two subgroups existed, one represented by NO_2_–N, and the other by the remaining physicochemical parameters. With regard to points, one group consisted of Points 3 and 14, and the other had the remaining points (within this group, 2 subgroups can be distinguished: one with Points 13 and 15, and another with others). The analysis confirmed that the quality of water in the Turawa reservoir was strongly influenced by Points 3 and 14 and, to a lesser extent, Points 13 and 15. With regard to the parameters, relationships were not visible (pH and NO_2_–N were not responsible for the deterioration of water quality, considering the results of water quality indices and ecological status).

#### Principal component analysis (PCA)

**Figure 17:**
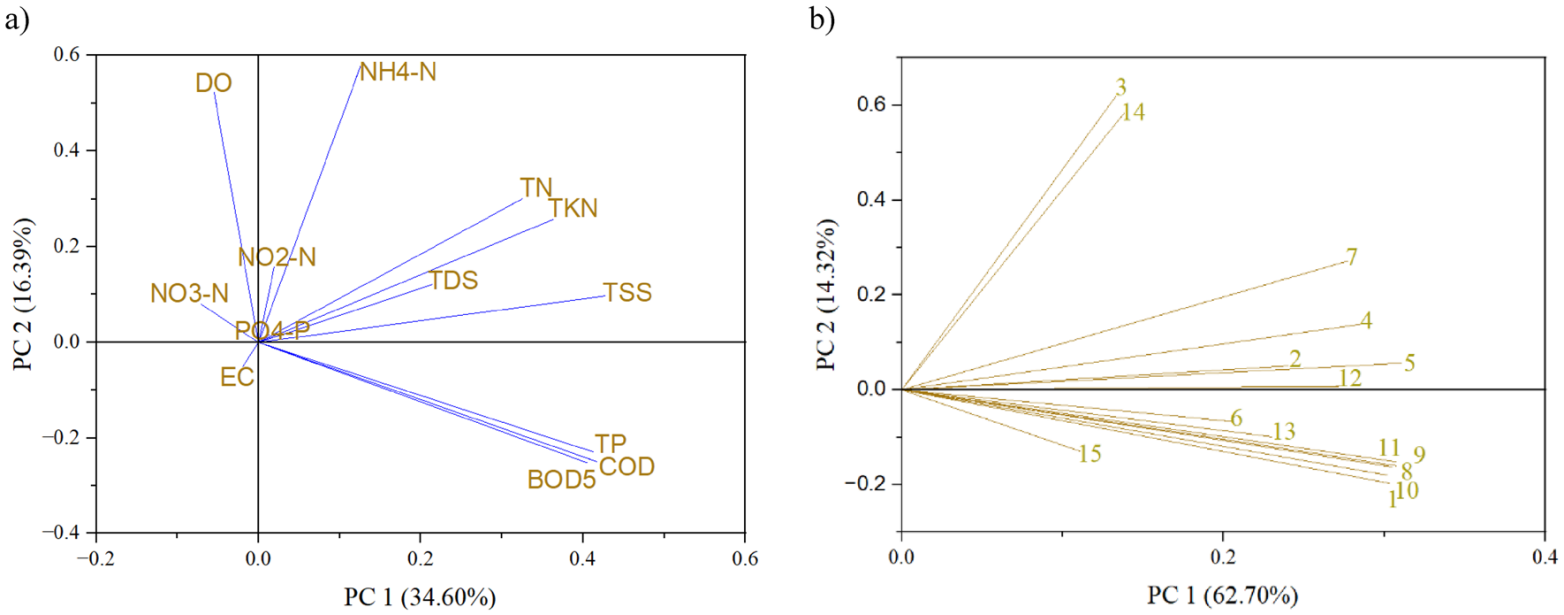
Principal Component Analysis (PCA) for the results of the (**a**) physicochemical parameters of water and (**b**) research points of the Turawa reservoir in 2019–2020.

The Bartlett's sphericity test performed for the PCA indicates the statistical significance of the data for *p* < 0.05, and the communalities, indicating the usefulness of all parameters for further analysis, except for pH (value after extraction equals 0.263). After this verification, the average value for the parameters after extraction was 0.824 (values from 0.660 for TDS to 0.929 for COD), so the extracted 5 components (eigenvalue > 1.0, explaining 82.42% of cases) were adequately represented by the data. As regards the parameters, most of them were highly correlated with the first (TN, TKN, TP, BOD_5_, COD, and TSS); second (NH_4_–N and DO); third (NO_2_–N and PO_4_–P); fourth (NO_3_–N); and fifth (EC and TDS) components. The spatial representation of the results in Fig. 17 confirms these relationships. Most of the parameters that were strongly correlated with the first and second components were responsible for the deterioration of water quality, as also expressed in water quality indices and ecological status, as described earlier (i.e., TP, BOD_5_, COD, and DO). Distinguishing as many as 5 components indicates, however, that the very variability of parameters is wide and they affect the water quality of the Turawa reservoir in different ways, and their analysis is complex and multidimensional. When analyzing the PCA for the points, a clear distinction was observed among Points 3, 14, and 15 on the graph, which were the furthest from the other points on the X axis and explained 62.70% of cases. Additionally, these points were the most responsible for the water quality deterioration in the Turawa water reservoir in the context of assessing compliance with the standards for ecological status. The performed PCA can be a tool for identifying and analyzing both the sources of water pollution and their potential interactions (physicochemical parameters), as well as informing about the location of these pollutants where corrective actions should be taken.

#### Spearman’s correlation matrix

**Figure 18:**
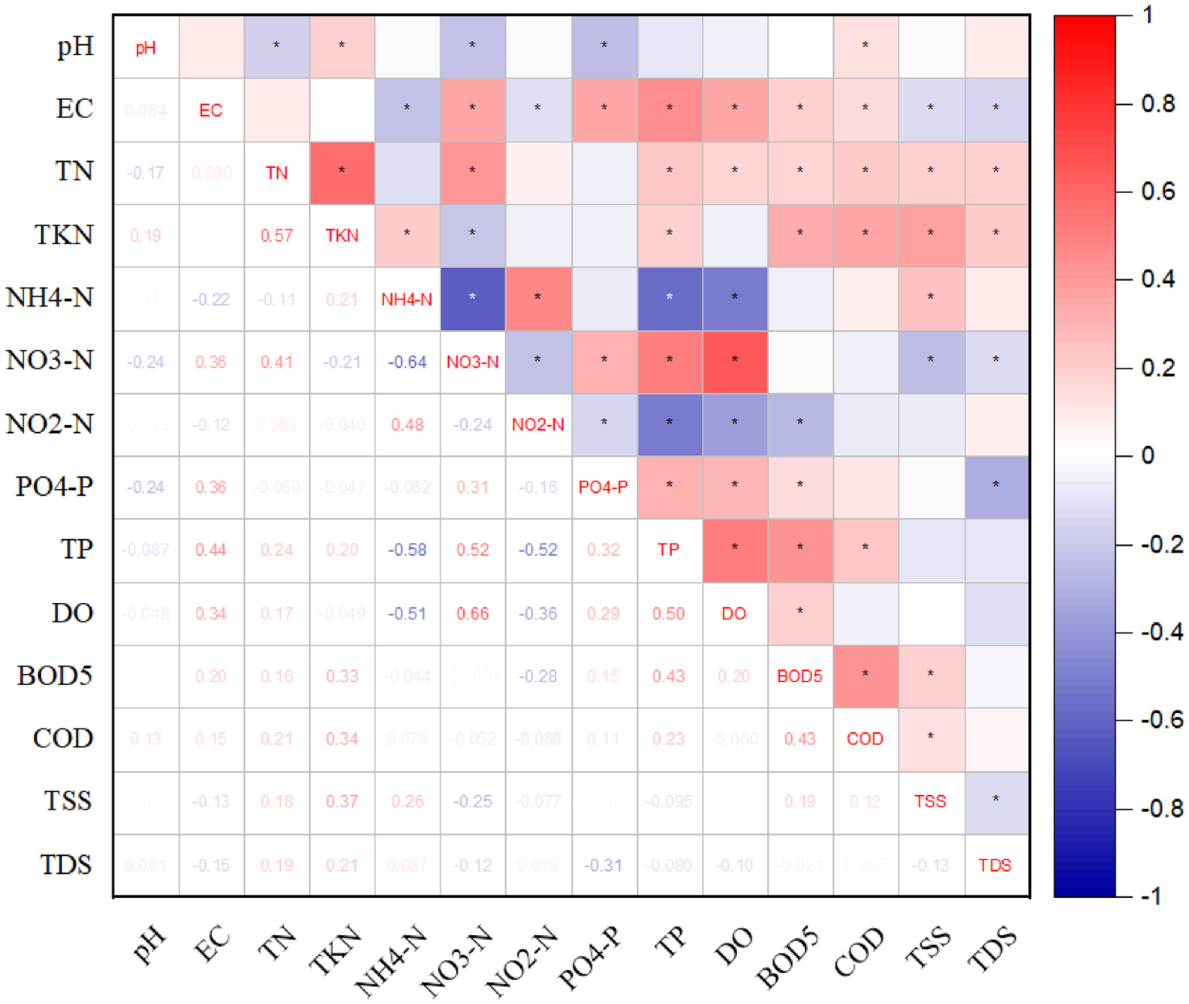
Spearman's correlation matrix for the results of physicochemical parameters of water of the Turawa reservoir in 2019–2020, where, * is the significant mark for the significant level (*p* < 0.05).

The Spearman correlation matrix for the points (Fig. 18) did not show strong correlation and statistically significant relationships between the points, and therefore, be omitted. Regarding the parameters, the statistical significance of the results appeared many times (for *p* < 0.05); however, the correlation strength was not too high. The maximum statistically significant relationship was obtained for the following pairs of parameters (R = 0.5–0.7 indicates moderate correlation): NO_3_–N and DO (0.66), NH_4_–N and NO_3_–N (− 0.64), TP and NH_4_–N (− 0.58), TKN and TN (0.57), NO_3_–N and TP (0.52), DO and NH_4_–N (− 0.51), and TP and DO (0.50). Ranking the results by their statistical significance between the pairs of parameters in descending order, the sequence was: NO_3_–N (11) > TP/EC (10) > TN/TKN/BOD_5_ (9) > PO_4_–P/TSS/DO (8) > NH_4_–N/NO_2_–N/COD (7) > TDS (6) > pH (5). The analysis confirmed that pH was the most outlier parameter.

## Discussion

The results show that the main problem of the Turawa reservoir is eutrophication, which manifests itself especially in increased concentrations of TP, BOD_5_, and COD and reduced concentrations of DO. This is in line with the research by Wiatkowski and Czerniawska-Kusza (2009)^73^ and Gruss et al. (2021)^43^, who indicated that the water analyzed in the Turawa reservoir in spring and summer, as a result of rising temperatures and increased pH, was characterized by the release of phosphorus from deeper sediment layers to the surface water. This tendency was also confirmed by the research of Hu et al. (2019)^74^ on the Biliuhe reservoir in China and Moura et al. (2020)^75^ on three water bodies in Brazil.

The results obtained by other researchers confirm that the intensification of the eutrophication process and changes in the water quality of water reservoirs (expressed by various indices) are influenced by both natural (meteorological, geological, hydrological) and anthropogenic (agricultural, industrial and municipal) factors. They are summarized in Table 6.

**Table 6:** Comparison of selected indices of water quality and trophic status along with the characteristics of the results obtained by various researchers.

Index value	Location	Characteristics of the results	Reference
TSI			
45–72	South Korea (60 reservoirs, monthly data from 2015–2017)	Different values of TSI, depending on the type of reservoir (descending): estuarine (hypereutrophic) > power generation (eutrophic) > natural lakes (eutrophic) > agricultural (mesotrophic) > multipurpose (mesotrophic)	Mamun et al., 2020^76^
47–67	Tucuruí reservoir, Brazil (2009–2012)	Mesotrophic or eutrophic, modified by human activity and sedimentation in the reservoir; the lowest values in the emptying phase of the reservoir the highest in the filling phase	da Costa Lobato et al., 2015^77^
57–81	Tri An Reservoir, Vietnam (2018–2019, bi-monthly data)	Light-eutrophic to hypereutrophic; condition caused by intense algae blooms, especially in April and June (preventing the function of the reservoir as a source of drinking water)	Pham et al., 2022^78^
42–66	Brazil (6 reservoirs in São Paulo State, 2010–2019)	Mesotrophic to hypereutrophic; a condition caused by the lack of order in water and sewage management and runoff of agricultural origin, carrying large loads of nutrients; Higher water temperatures were the factor intensifying the growth of algae	Cunha et al., 2021^79^
58–80	Tiru Reservoir, India (2017–2018)	Eutrophic to hypereutrophic; deterioration of the trophic state as a result of sediment transport by monsoons and agricultural runoff causing turbidity, less impact of algae	Markad et al., 2019^80^
50–78	Bhindawas Lake, India (2014)	Eutrophic to hypereutrophic; the greatest impact on the trophic state of total phosphorus from the resuspension of sediments during rains and strong winds, as well as from runoff from the surrounding agricultural areas	Saluja & Garg, 2017^81^
31–66	Poland (12 lakes in Suwałki Landscape Park, 1983, 1985, 2003, 2009)	Mesotrophic to eutrophic; the trophic state is influenced by the water exchange rate, the geological structure of the land and the way of use; the impact of phosphorus on the trophic state is low due to its inaccessible form, resulting from the properties of the soils in the catchment area (N is the limiting factor)	Jekaterynczuk-Rudczyk et al., 2014^82^
50–70	Sri Lanka (10 reservoirs, Kala Oya River Basin, 2013–2016)	Eutrophic; the limiting factor was the limitation of light access by particulate matter other than algae, and algae growth was affected by factors other than total phosphorus supplies; the supply of nutrients was inversely affected by water fluctuations in reservoirs	Nadarajah et al., 2019^83^
29–75	Narochanskie lakes, Belarus (1978–2013)	Oligotrophic to hypereutrophic; trophic status has improved over time; a high correlation was found between TSI and the content of sulfur and organic matter as well as BOD5 values; no correlation between TSI and TP content and the number of phytoplankton cells	Adamovich et al., 2016^84^
TLI			
10–90	Honghu lake, China (2000–2021)	From mesotrophic to eutrophic, variability in years; intensification of the eutrophication process in the summer months; factors affecting the intensity of eutrophication: air temperature, atmospheric precipitation, wind speed (higher temperatures and stagnant water favor the growth of algae), domestic sewage, industrial wastewater, intensive agricultural activities (fertilizers), climate change (increasing air temperature causes better conditions for the development algae), aquaculture (source of high loads of phosphorus)	Yang et al., 2023^85^
NSFWQI			
37–55	Lake Poyang, China (2009–2014)	Mostly moderate or good; deterioration over time to a low and bad state; the greatest impact on the deterioration of results on the part of TN and TP (83.06 and 70.21); it was also indicated that the problem of nutrient enrichment concerns other reservoirs in China—Dongting, Taihu, Hongze, Chaohu	Wu et al., 2017^86^
17.8–77.8	China (5 reservoirs in Yellow River catchment, 2007–2012)	From good to very poor water quality; increased Hg content caused deterioration of water quality; the deterioration of water quality in reservoirs is mainly due to agricultural activities and sources of municipal pollution	Hou et al., 2015^87^
Other WQI (weighted arithmetic index method)			
99.1–174.9	Koudiat Medouar reservoir, Algieria	From very poor to bad water quality; in most cases, the water is unfit for consumption, even after disinfection; the deterioration of the index results is due to high salinity, sulphates, low amounts of dissolved oxygen and high turbidity of the reservoir; the reason is anthropogenic impacts related to the discharge of municipal and industrial sewage, the lack of a proper sanitation system, agricultural runoff or the discharge of solid waste by local communities	Bouslah et al., 2017^88^

Progressing climate change (manifested by higher air temperatures and more frequent occurrence of torrential rains) is one of the factors influencing the intensification of the eutrophication process, which may result in more frequent occurrence of hydrological extreme phenomena such as floods and droughts. In the study area, floods occurred in 1997 and 2010, and were caused by heavy rainfall in mountainous areas, which was further manifested by rapid surface runoff moving downstream, carrying large loads of organic matter and nutrients^89–91^.

Additionally, studies on the water quality of the Mała Panew River and Turawa reservoir conducted in 1998–2009 and 2011–2016, respectively, indicate that the Turawa reservoir had an impact on improving the water quality of the Mała Panew River. The water in the reservoir exceeded the class II limit values for the following indicators: water temperature, TSS, pH, BOD_5_, NH_4_, TN, PO_4_, TP, and chlorides^42^.

As reflected by the results, Turawa reservoir belongs to a heavily modified part of water in poor condition, which is at risk of failing to achieve the assumed environmental goals. This condition consists of exceeding the standards for physicochemical elements (especially nutrients) and a chemical condition below good (exceeding Cd concentrations)^92^. This is mainly because of eutrophication, which is caused by the supply of biogenic compounds from municipal sources (mainly sewage and waste from nearby summer cottages and by tourists, as well as fertilizers and plant protection products used in agriculture), which are consumed by blue-green algae for their strong development in the water column (e.g., algae blooms)^93^. Water blooms cause deterioration of oxygen in the water and impart toxicity to people (e.g., bathers)^94,95^ and indicate the accumulation of heavy metals in the Turawa reservoir, such as Cu, Zn, Cd, and Pb in *Spirogyra sp.* algae and accumulated in bottom sediments^96^. Blooms also worsen the taste of caught fish, which can also be toxic due to high concentrations of cadmium in the water (ichthyofauna have a strong tendency to incorporate heavy metals into their own tissues and magnify, i.e., an increase in the concentration of these elements with an increase in the position in the trophic)^97^. Therefore, the reservoir has a problem with its assumed functioning during the growing seasons, both in terms of meeting the requirements for the protected area and its natural functions, as well as some recreational functions. Furthermore, the high intensity of algae growing on the water reservoir may pose a threat to the proper operation of water devices that control the levels and volume of water in the reservoir to meet the needs of water management^98^.

For prevention against eutrophication, in the water management plan for the Odra (Oder) basin in 2015–2022, it was decided to revitalize the Jedlice pre-reservoir (a bio-preparation was used to protect bottom sediments and a layer with a chemically active sorbent was placed on them). However, despite the implementation of the project, the Turawa reservoir still had functional issues. It should be mentioned that due to the failure to achieve the environmental objectives, the deadline for their implementation was postponed to the next planning cycle for 2016–2021. Due to technological limitations, other ways of counteracting the eutrophication process are currently being sought with low cost and high efficiency^99,100^.

Cheney water reservoir in the United States (Kansas), which has been supplying water to the population of Wichita since 1965, is an example of a water body that is majorly suffering from eutrophication due to cyanobacteria. However, the reservoir provides protection against flooding, scope of recreation, and a habitat for various groups of organisms^101^. The main reason for this phenomenon is the composition of bottom sediments (primarily silt and clay) and agricultural use of the catchment (plant protection products and animal products are sources of nutrients)^102^. These favorable conditions for the development of algae worsen living conditions for other organisms and especially the main function of the reservoir by deteriorating the quality of drinking water (exceeding the safe values for microcystins and geosmin)^103,104^. Therefore, models identifying the causes of eutrophication are created, documents related to water management on the reservoir and in the catchment are improved, water quality is constantly monitored, and proposals for remedial actions are developed^105,106^. The most important measures to manage water reservoir are to reduce the nutrient concentration (e.g. by using algae-eating zooplankton or more economical plant protection products) and limit bottom sediment accumulation (e.g. by removing bottom sediment from the reservoir and by developing an appropriate warning system before floods; 41% of the volume of accumulated sediments in 1966–2013 was from 8 days of flooding)^107,108^.

For many years, efforts have been made to retain fertilizing substances at the inflow to retention reservoirs^109,110^. As reported by Czamara et al. (2008)^111^, Wiatkowski, Rosik-Dulewska and Tymiński (2010)^112^, Wiatkowski and Rosik-Dulewska (2015)^113^, and Rinke et al. (2013)^114^, retention of fertilizing substances can be done using two methods: organizing water and sewage management in the catchment area, and building preliminary reservoirs. However, the former method provides the best results^115–117^, but is an expensive and long-term activity. The later method, using pre-reservoirs, is cheaper and faster to implement^118^. It mainly retains biogenic substances, drags, and floaters while also securing the main reservoir against emergency discharges of sewage and other substances, thereby creating additional water storage in the pre-reservoir^119,120^. Additionally, the following can be used: protective belts along watercourses and reservoirs; construction of girders to capture surface runoff; and appropriate use of agricultural land around reservoirs, near river beds, and even throughout the catchment area^121–123^. Chen et al. (2020)^124^ and Perrow et al. (1997)^125^, in addition to stopping the transport of hazardous substances through the reservoir bowl, also mention biomanipulation as a process supporting the reclamation of the reservoir. However, Zalewski et al. (2013)^126^, Tundisi & Tundisi (2016)^127^, and Paul et al. (2022)^128^ proposed the use of ecohydrology as an interdisciplinary tool for integrated water protection. For the reservoirs, research is being carried out on reclamation methods as treatments aiming at improving the water purity, e.g., the use of biostructures for water purification^129^, and the so-called methods of creating substrates for growing organisms to increase the effect of decomposition of organic matter, its sedimentation, and with it the depletion of water in biogenic substances (bio-hydro structures and foil substrates). Furthermore, attempts are being made to use reactive materials for reduction of phosphorus load in surface waters^130,131^. The technology of ion-exchange resins is also found applicable to reservoirs for the removal of biogenic compounds, where the installation for water quality improvement is part of the system, limiting the inflow of pollutants from the catchment area and thereby, extracting phosphorus and nitrogen compounds from the surface of the reservoir itself without harming the ecosystem^132^. The effectiveness of technologies and solutions aimed at reducing or eliminating eutrophication in reservoirs may vary depending on many factors, such as the size and type of reservoir, type of pollutants, applied technological solutions, costs and environmental and social effects. Mechanical treatment, hydrogen oxidation, use of microorganisms, use of artificial filter systems and improvement of sewage systems can be used to reduce eutrophication. The effectiveness of these solutions depends on many factors, such as the condition of the reservoir before the introduction of the solution, the type of contamination, its level and costs^133,134^.

Summarizing the considerations included in the discussion, the methods of water treatment in reservoirs to reduce or prevent eutrophication can be divided into chemical, physical, and biological methods^135,136^. Chemical methods are rarely used and treated as the last resort in the fight against water blooms, due to their possible negative environmental effects^137^; they may include herbicides and algaecides, salt injections for polluted bottom sediments, and copper sulfate^16,138^. Physical methods are based on the reduction of endogenous nutrients in reservoirs^139^, and may include mechanical agitation, deep aeration with air injections, mechanical removal of algae, and materials limiting the release of phosphorus compounds from sediments^31,140^. Biological methods are used to restore the ecological balance of the ecosystem by using various groups of organisms to remove, accumulate, or transform nutrients flowing into the reservoir (microorganisms, and aquatic plants and animals)^141^. These methods may include biofilm technologies, biomanipulation, constructed wetlands, and phytoremediation^142,143^.

Research on the water quality in reservoirs can have a significant impact on the development of water management policy and strategy in Poland, they are crucial for understanding what pollutants occur in waters, what are their sources and how they affect the environment and human and animal health. Based on the results of water quality tests of reservoirs, it is possible to determine what pollutants are present in the water and at what level, which allows to take action to improve water quality^144^. Implementation of appropriate environmental protection measures, such as sewage treatment plants or reduction of the amount of industrial waste discharged into water, will allow to reduce the degree of water pollution^145^. The results of water quality tests of reservoirs can also influence decision-making regarding the development of water tourism or fishing. In the event that tests show the presence of substances harmful to human or animal health, such as chemicals or bacteria, the water will not be safe to use, which will have a negative impact on the water tourism and fishing markets^133^. They can influence the development of global environmental policy and spatial planning decisions. In the event that studies show that water quality is poor and requires urgent action, the government may decide to introduce new regulations to improve water quality, such as raising environmental standards in the industrial sector or financing programs to modernize wastewater treatment plants^146^. They are essential for the development of an effective water management policy and strategy that aims to improve water quality, protect the environment and human and animal health and also influence factors such as the protection of the environment and human health, sustainable development, international cooperation, international politics and the fight against poverty^146^.

The research could be extended to include an analysis of factors affecting changes in water quality and eutrophication, e.g. natural, hydrological, meteorological factors or in connection with climate change. The scope of the research could also include the analysis of the quality of groundwater or bottom sediments. The results of the presented research would be difficult to compare with reservoirs of a different nature than the Turawa water reservoir, i.e. an artificial dam reservoir with a large catchment area, located in the lowlands of the temperate climate zone. Different actions can be taken to improve the ecological status of a reservoir, depending on the causes of the ecological problems that occur in a given reservoir. The most important are pollution control, pollution removal, water level control and habitat improvement. Evaluation of the effectiveness of activities related to the improvement of the ecological condition of the reservoir is very important. It can be carried out at different levels, depending on the objectives of the activities. Efficiency can be assessed on a microscopic scale (e.g. change in nutrient concentration in the reservoir) as well as on a macroscopic scale (e.g. improvement in the overall ecological status of the reservoir). The final assessment of the effectiveness of the measures should be based on field observations and measurements of nutrient concentrations, water quality, etc. It is also important to carry out pre- and post-implementation studies in order to accurately compare their effectiveness^146–148^.

## Conclusions

From the analysis, the following conclusions were formulated:

1. In 11 out of 14 assessed physicochemical parameters, statistical significance was demonstrated using the Mann–Whitney U test by comparing the results of 2019 and 2020. Considering the differences in the median between these years, the highest values were recorded for NO_3_–N (807.14%), DO (726.92%), TP (642.86%), and PO_4_–P (450.00%), and the largest differences between the means were obtained for NO_3_–N (336.85%), PO_4_–P (266.53%), NH_4_–N (− 98.21%), and DO (97.59%). For 8 physicochemical parameters (NO_3_–N, NO_2_–N, NH_4_–N, TKN, PO_4_–P, DO, TDS, and EC), a convergent direction of changes in the median and mean values between the two years and significance in the Mann–Whitney U test were found.
2. A spatio-temporal analysis of the physicochemical parameters of the water in Turawa reservoir showed a high susceptibility to eutrophication processes, particularly, due to the influence of phosphorus and nitrogen compounds coming from both areas with high tourist intensity and pollution flowing from the Mała Panew River (mainly of agricultural origin), which was retained in the preliminary reservoir in the south-eastern part of the Turawa reservoir. The highest average annual concentration values in 2019 and 2020 were recorded for most parameters in July and August, corresponding to the highest intensity of water blooms. While the lowest values of the tested parameters were observed in November and December, when the algae vegetation was significantly extinguished due to prevailing low temperatures and worse light conditions than in the summer months.
3. The parameters deteriorating the ecological status were TP, DO, BOD_5_, and COD (exceeding limit values by 338%, 76%, 62% and 25%, respectively). On analyzing the research points, the worst ecological status in relation to the limiting values, as confirmed by the performed statistical analyses (HCA and PCA), was achieved at Points 3, 14, and 15 (holiday resorts and areas used for the fisheries).
4. In the research period 1998–2020, the Turawa reservoir was classified into supereutrophic and eutrophic, based on the calculated Carlson and trophic level indices. By the Vollenweider and Kajak method, the dangerous load of phosphorus and total nitrogen was found to be exceeding each year, with TN having a greater potential to cause eutrophication than TP (exceedances against dangerous loads at an average level of 25.79 and 2.85, respectively). Summarizing the exceedance values for both nutrients, the highest risk of eutrophication was recorded in 1998 (35.98), 1999 (38.85), and 2001 (44.70), and the lowest in 2003 (18.67), 2014 (18.84), and 2019 (14.82). The causes of water blooms in this area were municipal sources, i.e., waste dumped by tourists and residents, and agriculture (surface runoff of fertilizers and plant protection products from the fields). Eutrophication has a serious impact on the functioning of organisms living in the water, as well as on the health of people using the reservoir (algae causing eutrophication are toxic). It was assumed that corrective actions were carried out for the revitalization of the pre-reservoir, but they were ineffective.
5. Considering the aggregate results of water quality indices for the period 1998–2020, the average water quality was in classes II or III (i.e., good or moderate water quality; values from 54.40 in 2019 to 83.40 in 2016; five-class scale, from 0 to 100 points). In the case of the NSF WQI, in 6 out of 17 years, the water quality was good (class II), and in the remaining 11 years, it was very good (class I). For OWQI, these values were already lower and had characteristics of class II or III. The weakest values were recorded for UWQI, i.e., within III, IV, or V water quality classes. There was a noticeable deterioration in each water quality index for the years 2016–2019/2020. The most common parameters responsible for lower values of water quality indices were TP, NO_3_ (NO_3_–N), and BOD_5_, the deterioration of which indicates the progressing eutrophication in the Turawa water reservoir.
6. A statistically significant, moderate correlation (R = 0.5–0.7) was found for the following parameter pairs: NO_3_–N and DO (0.66), NH_4_–N and NO_3_–N (− 0.64), TP and NH_4_–N (− 0.58), TKN and TN (0.57), NO_3_–N and TP (0.52), DO and NH_4_–N (− 0.51), and TP and DO (0.50). Ranking the results by the number of statistically significant results between pairs of parameters in descending order, the sequence was: NO_3_–N (11) > TP/EC (10) > TN/TKN/BOD_5_ (9) > PO_4_–P/TSS/DO (8) > NH_4_–N/NO_2_–N/COD (7) > TDS (6) > pH (5).
7. The water management plan stipulates that the Turawa reservoir is at risk, and therefore this status should be improved through the implementation of various projects. It was noted that in the current situation, technological implementation is difficult due to costs. Therefore, high-performance and low-cost technological solutions should be sought to prevent eutrophication. An example of such solutions to reduce or prevent eutrophication can be chemical, physical, and biological methods of water treatment (e.g., the use of herbicides, deep aeration with air injections, and biomanipulation), as well as retaining fertilizing substances at the inflow to retention reservoirs by organizing water and sewage management in the catchment and constructing preliminary reservoirs.
8. Future research should focus on examining the effectiveness of using various technologies that could reduce or eliminate eutrophication in the Turawa water reservoir (as well as other eutrophic reservoirs). Thanks to the study of the effectiveness of such solutions, an effective, long-term plan for the reconstruction of this reservoir could be developed, which would fulfill the assumed natural, social, and economic functions.
9. At the Turawa reservoir, it will be important to maintain an appropriate water level so as not to endanger aquatic life on the reservoir, and to control pollution by identifying its sources in the catchment area and limiting their inflow to the reservoir.

**Figure 19:**
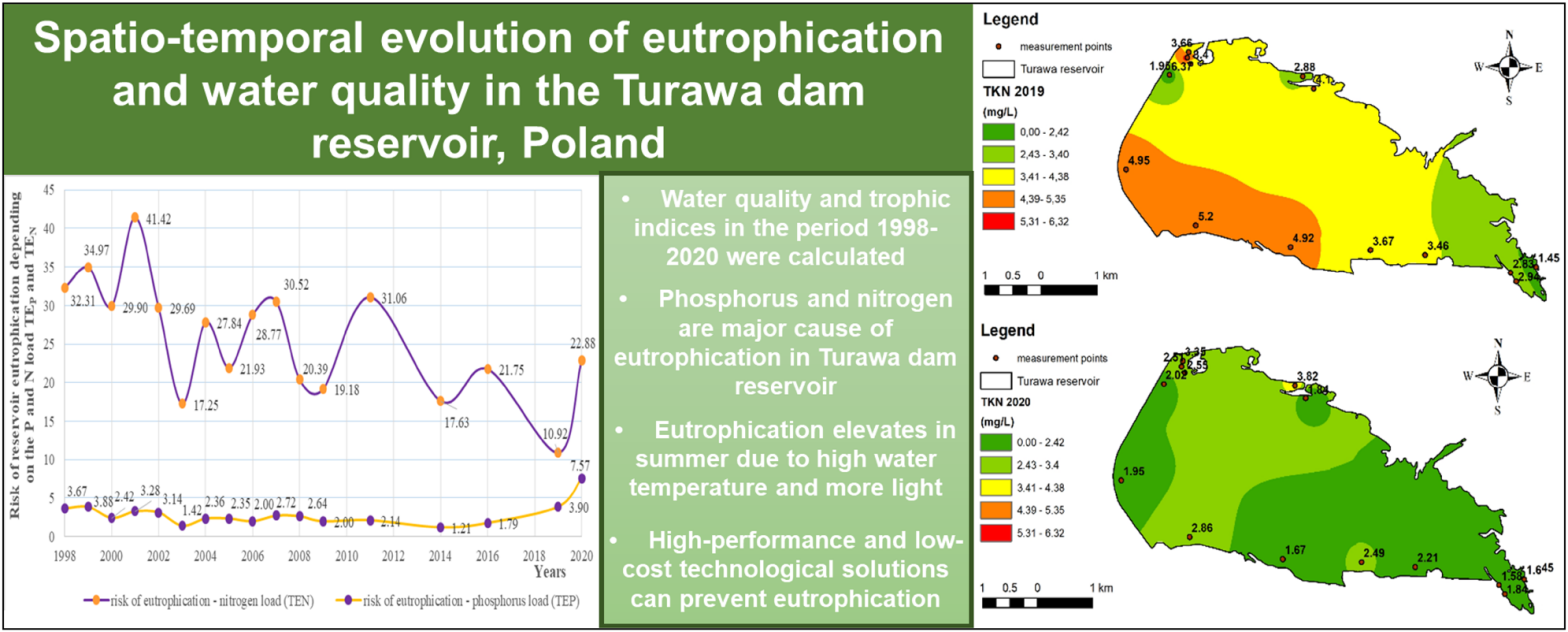
Graphical abstract (map generated in ArcGIS 10.5.1, background map: the 1:10,000 Map of the Hydrographic Division of Poland, interpolation: Inverse Distance Weighted method).

A graphical abstract summarizing the work done in the article is presented in the Fig. 19.

## Data Availability

All data generated or analysed during this study are included in this published article.

## Abbreviations

BOD_5_: Five-day biochemical oxygen demand
COD: Chemical oxygen demand
CH_4_: Methane
CO_2_: Carbon dioxide
DO: Dissolved oxygen
DWQI: Dilnius’ water quality index
EC: Electrical conductivity
HCA: Hierarchical cluster analysis
NH_4_: Ammonia
NH_4_–N: Ammonium nitrogen
NO_2_: Nitrites
NO_2_–N: Nitrite nitrogen
NO_3_: Nitrates
NO_3_–N: Nitrate nitrogen
NSF WQI: National sanitation foundation water quality index
OIP: Overall index pollution
OWQI: Oregon water quality index
PCA: Principal component analysis
PO_4_–P: Phosphate phosphorus
TDS: Total dissolved solids
TL_C_: Trophic level index for chlorophyll a
TLI: Trophic level index
TL_N_: Trophic level index for total nitrogen
TL_P_: Trophic level index for total phosphorus
TL_S_: Trophic level index for Secchi depth
TN: Total nitrogen
TP: Total phosphorus
TSI_P_: Carlson index (trophic state index)
TSS: Total suspended solids
WIOŚ: Province inspectorate for environmental protection
UWQI: Universal water quality index
